# Evidence-based recommendations for natural bodybuilding contest preparation: nutrition and supplementation

**DOI:** 10.1186/1550-2783-11-20

**Published:** 2014-05-12

**Authors:** Eric R Helms, Alan A Aragon, Peter J Fitschen

**Affiliations:** 1 Sport Performance Research in New Zealand (SPRINZ) at AUT Millennium Institute, AUT University, 17 Antares Place, Mairangi Bay, Auckland 0632, New Zealand; 2 California State University, Northridge, CA, USA; 3 Division of Nutritional Sciences, University of Illinois, Urbana, IL, USA

**Keywords:** Hypertrophy, Calories, Frequency, Nutrient, Body fat, Resistance training, Competition

## Abstract

The popularity of natural bodybuilding is increasing; however, evidence-based recommendations for it are lacking. This paper reviewed the scientific literature relevant to competition preparation on nutrition and supplementation, resulting in the following recommendations. Caloric intake should be set at a level that results in bodyweight losses of approximately 0.5 to 1%/wk to maximize muscle retention. Within this caloric intake, most but not all bodybuilders will respond best to consuming 2.3-3.1 g/kg of lean body mass per day of protein, 15-30% of calories from fat, and the reminder of calories from carbohydrate. Eating three to six meals per day with a meal containing 0.4-0.5 g/kg bodyweight of protein prior and subsequent to resistance training likely maximizes any theoretical benefits of nutrient timing and frequency. However, alterations in nutrient timing and frequency appear to have little effect on fat loss or lean mass retention. Among popular supplements, creatine monohydrate, caffeine and beta-alanine appear to have beneficial effects relevant to contest preparation, however others do not or warrant further study. The practice of dehydration and electrolyte manipulation in the final days and hours prior to competition can be dangerous, and may not improve appearance. Increasing carbohydrate intake at the end of preparation has a theoretical rationale to improve appearance, however it is understudied. Thus, if carbohydrate loading is pursued it should be practiced prior to competition and its benefit assessed individually. Finally, competitors should be aware of the increased risk of developing eating and body image disorders in aesthetic sport and therefore should have access to the appropriate mental health professionals.

## Introduction

The popularity of natural bodybuilding is increasing rapidly. In the United States, over 200 amateur natural (drug tested) bodybuilding contests occurred during 2013 and the number of contests is expected to increase in 2014 [1]. Preparation for bodybuilding competition involves drastic reductions in body fat while maintaining muscle mass. This is typically achieved through a decreased caloric intake, intense strength training, and increased cardiovascular exercise. Competitors partake in numerous dietary and supplementation strategies to prepare for a contest. Some have a strong scientific basis; however, many do not. Therefore, the purpose of this article is to review the scientific literature on topics relevant to nutrition and supplementation for bodybuilding competition preparation. Dietary modifications during the last week to enhance muscle definition and fullness (peaking) and psychosocial issues will also be covered. Ultimately, evidence-based recommendations will be made for nutrition, supplementation, and “peak week” strategies for natural bodybuilders. As a final note, this paper does not cover training recommendations for natural bodybuilding and the training methodology used will interact with and modify the effects of any nutritional approach.

## Methods

PubMed, MEDLINE, SPORTDiscus and CINAHL electronic databases were searched online. Each author was assigned a portion of the manuscript to write specific to their area(s) of expertise. Authors performed searches for key words associated with their portion(s) of the manuscript; calories and macronutrients, nutrient timing and meal frequency, dietary supplementation, psychosocial issues and “peak week” were the selected topics. The publications obtained were carefully screened for studies that included healthy humans or humans in a caloric deficit. Long-term human studies focusing on hypertrophy and body fat loss were preferentially selected; however, acute studies and/or studies using animal models were selected in the absence of adequate long-term human studies. In addition, author names and reference lists were used for further search of the selected papers for related references. As this review is intended to be an evidence-based guide and the available data relevant to natural bodybuilding is extremely limited, a narrative review style was chosen.

### Nutrition

#### Calories and macronutrients

Competitive bodybuilders traditionally follow two to four month diets in which calories are decreased and energy expenditure is increased to become as lean as possible [2-6]. In addition to fat loss, muscle maintenance is of primary concern during this period. To this end, optimal caloric intakes, deficits and macronutrient combinations should be followed while matching the changing needs that occur during competition preparation.

### Caloric intake for competition

To create weight loss, more energy must be expended than consumed. This can be accomplished by increasing caloric expenditure while reducing caloric intake. The size of this caloric deficit and the length of time it is maintained will determine how much weight is lost. Every pound of pure body fat that is metabolized yields approximately 3500 kcals, thus a daily caloric deficit of 500 kcals theoretically results in fat loss of approximately one pound per week if the weight loss comes entirely from body fat [7]. However, a static mathematical model does not represent the dynamic physiological adaptations that occur in response to an imposed energy deficit [8]. Metabolic adaptation to dieting has been studied in overweight populations and when observed, reductions in energy expenditure amount to as little as 79 kcal/d [9], to as much as 504 kcal/d beyond what is predicted from weight loss [10]. Metabolic adaptations to bodybuilding contest preparation have not been studied however; non-overweight men who consumed 50% of their maintenance caloric intake for 24 weeks and lost one fourth of their body mass experienced a 40% reduction in their baseline energy expenditure. Of that 40% reduction 25% was due to weight loss, while metabolic adaptation accounted for the remaining 15% [11]. Therefore, it should be expected that the caloric intake at which one begins their preparation will likely need to be adjusted over time as body mass decreases and metabolic adaptation occurs. A complete review of metabolic adaptation to dieting in athletes is beyond the scope of this review. However, coaches and competitors are encouraged to read the recent review on this topic by Trexler et al. [12] which covers not only the physiology of metabolic adaptation, but also potential methods to mitigate its negative effects.

In determining an appropriate caloric intake, it should be noted that the tissue lost during the course of an energy deficit is influenced by the size of the energy deficit. While greater deficits yield faster weight loss, the percentage of weight loss coming from lean body mass (LBM) tends to increase as the size of the deficit increases [7,13-15]. In studies of weight loss rates, weekly losses of 1 kg compared to 0.5 kg over 4 weeks resulted in a 5% decrease in bench press strength and a 30% greater reduction in testosterone levels in strength training women [16]. Weekly weight loss rates of 1.4% of bodyweight compared to 0.7% in athletes during caloric restriction lasting four to eleven weeks resulted in reductions of fat mass of 21% in the faster weight loss group and 31% in the slower loss group. In addition, LBM increased on average by 2.1% in the slower loss group while remaining unchanged in the faster loss group. Worthy of note, small amounts of LBM were lost among leaner subjects in the faster loss group [13].

Therefore, weight loss rates that are more gradual may be superior for LBM retention. At a loss rate of 0.5 kg per week (assuming a majority of weight lost is fat mass), a 70 kg athlete at 13% body fat would need to be no more than 6 kg to 7 kg over their contest weight in order to achieve the lowest body fat percentages recorded in competitive bodybuilders following a traditional three month preparation [4,6,17-20]. If a competitor is not this lean at the start of the preparation, faster weight loss will be required which may carry a greater risk for LBM loss.

In a study of bodybuilders during the twelve weeks before competition, male competitors reduced their caloric intake significantly during the latter half and subsequently lost the greatest amount of LBM in the final three weeks [21]. Therefore, diets longer than two to four months yielding weight loss of approximately 0.5 to 1% of bodyweight weekly may be superior for LBM retention compared to shorter or more aggressive diets. Ample time should be allotted to lose body fat to avoid an aggressive deficit and the length of preparation should be tailored to the competitor; those leaner dieting for shorter periods than those with higher body fat percentages. It must also be taken into consideration that the leaner the competitor becomes the greater the risk for LBM loss [14,15]. As the availability of adipose tissue declines the likelihood of muscle loss increases, thus it may be best to pursue a more gradual approach to weight loss towards the end of the preparation diet compared to the beginning to avoid LBM loss.

#### Determining macronutrient intake

##### Protein

Adequate protein consumption during contest preparation is required to support maintenance of LBM. Athletes require higher protein intakes to support increased activity and strength athletes benefit from higher intakes to support growth of LBM [5,22-28]. Some researchers suggest these requirements increase further when athletes undergo energy restriction [13,16,22,28-33]. Furthermore, there is evidence that protein requirements are higher for leaner individuals in comparison to those with higher body fat percentages [7,33,34].

The collective agreement among reviewers is that a protein intake of 1.2-2.2 g/kg is sufficient to allow adaptation to training for athletes whom are at or above their energy needs [23-28,35-38]. However, bodybuilders during their contest preparation period typically perform resistance and cardiovascular training, restrict calories and achieve very lean conditions [2-6,17-21]. Each of these factors increases protein requirements and when compounded may further increase protein needs [33]. Therefore, optimal protein intakes for bodybuilders during contest preparation may be significantly higher than existing recommendations.

In support of this notion, Butterfield et al. [22] found that male athletes running five to 10 miles per day during a slight caloric deficit were in a significant negative nitrogen balance despite consuming 2 g/kg of protein daily. Celejowa et al. [39] showed that five out of 10 competitive weight lifters achieved a negative nitrogen balance over the course of a training camp while consuming an average protein intake of 2 g/kg. Out of these five, as many as three were in a caloric deficit. The authors concluded that a protein intake of 2–2.2 g/kg under these conditions only allows for a small margin of error before nitrogen losses occur.

Walberg et al. [32] examined the effects of two energy restricted isocaloric diets of differing protein intakes in 19 lean (9.1-16.7% body fat), male, non-competitive body builders. One group consumed a protein intake of 0.8 g/kg and higher carbohydrates, while the other consumed 1.6 g/kg of protein with lower carbohydrates. The length of the intervention was only one week, but nonetheless nitrogen losses occurred only in the lower protein group and LBM decreased by a mean of 2.7 kg in the 0.8 g/kg protein group and by a mean of 1.4 kg in the 1.6 g/kg protein group. While the high protein group mitigated LBM losses compared to the low protein group, they were not eliminated.

A recent study by Mettler et al. [29] employed the same basic methodology as Walberg et al. [32]. However, one group consumed a protein intake of 1 g/kg, while the other consumed 2.3 g/kg. The high-protein group lost significantly less LBM (0.3 kg) over the course of the two week intervention compared to the low-protein group (1.6 kg). Unlike Walberg et al. [32] calorie balance between diets was maintained by reducing dietary fat as opposed to carbohydrate to allow for the increase in protein.

While it appears that the 2.3 g/kg protein intervention in Mettler et al. [29] was superior for maintaining LBM compared to 1.6 g/kg in Walberg et al. [32] a recent study by Pasiakos et al. [40] found a trend towards the opposite. In this study, a non-significant trend of greater LBM retention occurred when subjects consumed 1.6 g/kg of protein compared to 2.4 g/kg of protein. However, the participants were intentionally prescribed low volume, low intensity resistance training "to minimize the potential of an unaccustomed, anabolic stimulus influencing study outcome measures". Thus, the non-anabolic nature of the training may not have increased the participants’ protein requirements to the same degree as the participants in Mettler et al. [29] or to what would be expected among competitive bodybuilders.

Maestu et al. [6] did not observe a significant loss of LBM in a group of drug free bodybuilders consuming 2.5-2.6 g/kg of protein during the 11 weeks prior to competition. These results when considered alongside the works by Walberg et al. [32] and Mettler et al. [29] imply that the higher the protein intake, the lower the chance for LBM loss. However, it should be noted that this study did not include a low protein control and not all studies show a linear increase in LBM preservation with increases in protein [40]. Furthermore, two subjects did lose significant amounts of LBM (1.5 kg and 1.8 kg), and the authors noted that these specific bodybuilders were among the leanest of the subjects. These two subjects lost the majority of their LBM (approximately 1 kg) during the latter half of the intervention as their percentage of calories from protein increased from 28% to 32-33% by the end of the study. The group as a whole progressively decreased their calories by reducing all three macronutrients throughout the investigation. Thus, the two subjects uniquely increased their proportion of protein, possibly reducing fat and carbohydrate to the point of detriment [6]. That said it is also plausible that the lost LBM seen by these two subjects was necessary in order to achieve their low levels of body fat. It is unknown whether or not the lost LBM influenced their competitive outcome and it is possible that had the competitors not been as lean, they may have retained more LBM but also not have placed as well.

In a review by Phillips and Van Loon [28], it is suggested that a protein intake of 1.8-2.7 g/kg for athletes training in hypocaloric conditions may be optimal. While this is one of the only recommendations existing that targets athletes during caloric restriction, this recommendation is not given with consideration to bodybuilders performing concurrent endurance and resistance training at very low levels of body fat. However, the recently published systematic review by Helms et al. [33] on protein intakes in resistance-trained, lean athletes during caloric restriction suggests a range of 2.3-3.1 g/kg of LBM, which may be more appropriate for bodybuilding. Moreover, the authors suggest that the lower the body fat of the individual, the greater the imposed caloric deficit and when the primary goal is to retain LBM, the higher the protein intake (within the range of 2.3-3.1 g/kg of LBM) should be.

##### Carbohydrate

High carbohydrate diets are typically thought to be the athletic performance standard. However, like protein, carbohydrate intake needs to be customized to the individual. Inadequate carbohydrate can impair strength training [41] and consuming adequate carbohydrate prior to training can reduce glycogen depletion [42] and may therefore enhance performance.

While it is true that resistance training utilizes glycogen as its main fuel source [43], total caloric expenditure of strength athletes is less than that of mixed sport and endurance athletes. Thus, authors of a recent review recommend that carbohydrate intakes for strength sports, including bodybuilding, be between 4–7 g/kg depending on the phase of training [26]. However, in the specific case of a bodybuilder in contest preparation, achieving the necessary caloric deficit while consuming adequate protein and fat would likely not allow consumption at the higher end of this recommendation.

Satiety and fat loss generally improve with lower carbohydrate diets; specifically with higher protein to carbohydrate ratios [44-49]. In terms of performance and health, low carbohydrate diets are not necessarily as detrimental as typically espoused [50]. In a recent review, it was recommended for strength athletes training in a calorically restricted state to reduce carbohydrate content while increasing protein to maximize fat oxidation and preserve LBM [28]. However, the optimal reduction of carbohydrate and point at which carbohydrate reduction becomes detrimental likely needs to be determined individually.

One comparison of two isocaloric, energy restricted diets in bodybuilders showed that a diet that provided adequate carbohydrate at the expense of protein (1 g/kg) resulted in greater LBM losses compared to a diet that increased protein (1.6 g/kg) through a reduction of carbohydrate [32]. However, muscular endurance was degraded in the lower carbohydrate group. In a study of athletes taking in the same amount of protein (1.6 g/kg) during weight loss, performance decrements and LBM losses were avoided when adequate carbohydrate was maintained and dietary fat was lowered [13]. Mettler, et al. [29] also found that a caloric reduction coming from dietary fat while maintaining adequate carbohydrate intake and increasing protein to 2.3 g/kg maintained performance and almost completely eliminated LBM losses in resistance trained subjects. Finally, in Pasiakos et al. [40] participants undergoing an equal calorie deficit and consuming the same amount of protein as those observed in Mettler et al. [29] lost three times the amount of LBM over the same time period (0.9 kg in the first two weeks of energy restriction observed by Pasiakos versus 0.3 kg observed by Mettler). One key difference between these studies was the highest protein group in Mettler et al. [29] consumed a 51% carbohydrate diet while the comparable group in Pasiakos et al. [40] consumed a 27% carbohydrate diet. While performance was not measured, the participants in Pasiakos et al. [40] performing sets exclusively of 15 repetitions very likely would have experienced decrements in performance due to this carbohydrate intake level [32]. The difference in training protocols or a nutritionally mediated decrement in training performance could have either or both been components that lead to the greater losses of LBM observed by Pasiakos et al. [40].

While it appears low carbohydrate, high protein diets can be effective for weight loss, a practical carbohydrate threshold appears to exist where further reductions negatively impact performance and put one at risk for LBM losses. In support of this notion, researchers studying bodybuilders during the final 11 weeks of contest preparation concluded that had they increased carbohydrate during the final weeks of their diet they may have mitigated metabolic and hormonal adaptations that were associated with reductions in LBM [6].

Therefore, once a competitor has reached or has nearly reached the desired level of leanness, it may be a viable strategy to reduce the caloric deficit by an increase in carbohydrate. For example, if a competitor has reached competition body fat levels (lacking any visible subcutaneous fat) and is losing half a kilogram per week (approximately a 500 kcals caloric deficit), carbohydrate could be increased by 25-50 g, thereby reducing the caloric deficit by 100-200 kcals in an effort to maintain performance and LBM. However, it should be noted that like losses of LBM, decrements in performance may not affect the competitive outcome for a bodybuilder. It is possible that competitors who reach the leanest condition may experience unavoidable drops in performance.

##### Fat

The importance of carbohydrate and protein in sports nutrition is often emphasized over that of dietary fat. Subsequently, recommendations typically focus on maintaining adequate fat intake while emphasizing carbohydrate to fuel performance and protein to build and repair LBM. However, there is evidence that dietary fat influences anabolic hormone concentrations which may be of interest to bodybuilders attempting to maintain LBM while dieting [5,26,51,52].

Reductions in the percentage of dietary fat in isocaloric diets from approximately 40% to 20% has resulted in modest, but significant, reductions in testosterone levels [53,54]. However, distinguishing the effects of reducing total dietary fat on hormonal levels from changes in caloric intake and percentages of saturated and unsaturated fatty acids in the diet is difficult [51,52,55]. In a study by Volek et al. [51], correlations were found between testosterone levels, macronutrient ratios, types of lipids, and total dietary fat, illustrating a complex interaction of variables. In a similar study of resistance trained males, correlations were found between testosterone, protein, fat and saturated fat which lead the researchers to conclude that diets too low in fat or too high in protein might impair the hormonal response to training [52].

Competing bodybuilders must make an obligatory caloric reduction. If a reduction in fat is utilized, it may be possible to attenuate a drop in testosterone by maintaining adequate consumption of saturated fat [5]. However, a drop in testosterone does not equate to a reduction in LBM. In direct studies of resistance trained athletes undergoing calorically restricted high protein diets, low fat interventions that maintain carbohydrate levels [13,29] appear to be more effective at preventing LBM loses than lower carbohydrate, higher fat approaches [32,40]. These results might indicate that attempting to maintain resistance training performance with higher carbohydrate intakes is more effective for LBM retention than attempting to maintain testosterone levels with higher fat intakes.

Body composition and caloric restriction may play greater roles in influencing testosterone levels that fat intake. During starvation, a reduction in testosterone occurs in normal weight, but not obese, males [56]. In addition, rate of weight loss may influence testosterone levels. Weekly target weight loss rates of 1 kg resulted in a 30% reduction in testosterone compared to target weight loss rates of 0.5 kg per week in resistance trained women of normal weight [16]. Additionally, an initial drop in testosterone occurred in the first six weeks of contest preparation in a group of drug free bodybuilders despite various macronutrient percentages [6]. Finally, in a one year case study of a natural competitive bodybuilder, testosterone levels fell to one fourth their baseline values three months into the six month preparation period. Levels then fully recovered three months into the six month recovery period. Testosterone did not decline further after the initial drop at the three month mark despite a slight decrease in fat intake from 27% to 25% of calories at the six month mark. Furthermore, the quadrupling of testosterone during the recovery period from its suppressed state back to baseline was accompanied by a 10 kg increase in body mass and a 1000 kcal increase in caloric intake. However, there was only a minor increase in calories from fat (percentage of calories from fat during recovery was between (30 and 35%) [57]. Finally, these testosterone changes in men appear mostly related to energy availability (body fat content and energy balance), and not surprisingly low-levels of sustained energy availability are also the proposed cause of the hormonal disturbance “athletic amenorrhea” in women [58]. Thus, the collective data indicates that when extremely lean body compositions are attained through extended, relatively aggressive dieting, the caloric deficit and loss of body fat itself may have a greater impact on testosterone than the percentage of calories coming from dietary fat.

While cogent arguments for fat intakes between 20 to 30% of calories have been made to optimize testosterone levels in strength athletes [59], in some cases this intake may be unrealistic in the context of caloric restriction without compromising sufficient protein or carbohydrate intakes. While dieting, low carbohydrate diets may degrade performance [32] and lead to lowered insulin and IGF-1 which appear to be more closely correlated to LBM preservation than testosterone [6]. Thus, a lower end fat intake between 15-20% of calories, which has been previously recommended for bodybuilders [5], can be deemed appropriate if higher percentages would reduce carbohydrate or protein below ideal ranges.

### Ketogenic diets and individual variability

Some bodybuilders do use very-low carbohydrate, "ketogenic diets" for contest preparation [60,61]. While these diets have not been sufficiently studied in bodybuilders, some study of ketogenic diets has occurred in resistance trained populations. In an examination of the effects of a 1 week ketogenic diet (5.4% of calories from carbohydrate) in subjects with at least 2 years of resistance training experience, Sawyer et al. [62] observed slight decreases in body fat among female participants and maintenance or slight increases in measures of strength and power among both male and female participants. However, it is difficult to draw conclusions due to the very short term nature of this study and due to an ad libitum implementation of the ketogenic diet. As implemented in this study, besides a reduction in carbohydrate and an increase in dietary fat, the ketogenic diet resulted in an average reduction of 381 calories per day and an increase of 56 g of protein per day compared to the participants’ habitual diets. Thus, it is unclear whether the improvements in body composition and performance can be attributed to the low-carbohydrate and high-fat nature of the diets or rather a decrease in calories and an increase in protein. At least with regards to weight loss, previous research indicates that the often concomitant increase in protein observed in very low carbohydrate diets may actually be the key to their success [63].

The only research on strength athletes following ketogenic diets for longer periods is a study of gymnasts in which they were observed to maintain strength performance and lose more body fat after 30 days on a ketogenic diet in comparison to 30 days on a traditional western diet [64]. However, this study's sample size was limited (n = 8) and it was not a controlled study of an intentional fat-loss phase such as seen among bodybuilders during competition preparation. Therefore, more study is needed in resistance trained populations and bodybuilders before definitive recommendations can be made to support ketogenic diets.

However, the research that does exist challenges traditional views on carbohydrate and anaerobic performance. Despite the common belief that carbohydrate is the sole fuel source for weight training, intramuscular triglyceride is used during short term heavy resistance training [65] and likely becomes an increasingly viable fuel source for those adapted to high-fat low-carbohydrate diets. While some might suggest that this implies a ketogenic diet could be a viable option for contest preparation, a trend of decreased performance and impaired maintenance of FFM is associated with lower carbohydrate intakes in the majority of studies included in this review.

While it is our contention that the majority of the evidence indicates that very-low carbohydrate diets should be avoided for contest preparation (at least until more research is performed), it must be noted that there is a high degree of variability in the way that individuals respond to diets. Carbohydrate and fat utilization as a percentage of energy expenditure at rest and various intensities has as much as a four-fold difference between individual athletes; which is influenced by muscle fiber-composition, diet, age, training, glycogen levels and genetics [66]. Additionally, individuals that are more insulin sensitive may lose more weight with higher-carbohydrate low-fat diets while those more insulin resistant may lose more weight with lower-carbohydrate higher-fat diets [67].

Due to this individual variability, some popular commercial bodybuilding literature suggests that somatotype and/or body fat distribution should be individually assessed as a way of determining macronutrient ratios. However, there is no evidence of any relationships with bone structure or regional subcutaneous fat distribution with any response to specific macronutrient ratios in bodybuilders or athletic populations. Bodybuilders, like others athletes, most likely operate best on balanced macronutrient intakes tailored to the energy demands of their sport [68].

In conclusion, while the majority of competitors will respond best to the fat and carbohydrate guidelines we propose, the occasional competitor will undoubtedly respond better to a diet that falls outside of these suggested ranges. Careful monitoring over the course of a competitive career is required to determine the optimal macronutrient ratio for pre-contest dieting.

#### Macronutrient recommendations summary

After caloric intake is established based on the time frame before competition [69], body composition of the athlete [14,15,34], and keeping the deficit modest to avoid LBM losses [13,16], macronutrients can be determined within this caloric allotment. Table 1 provides an overview of these recommendations.

**Table 1:** Dietary recommendations for bodybuilding contest preparation

**Diet component**	**Recommendation**
Protein (g/kg of LBM)	2.3-3.1 [33]
Fat (% of total calories)	15-30% [5,59]
Carbohydrate (% of total calories)	remaining
Weekly weight loss (% of body weight)	0.5-1% [13,16]

If training performance degrades it may prove beneficial to decrease the percentage of calories from dietary fat within these ranges in favor of a greater proportion of carbohydrate. Finally, while outside of the norm, some competitors may find that they respond better to diets that are higher in fat and lower in carbohydrate than recommended in this review. Therefore, monitoring of individual response over a competitive career is suggested.

#### Nutrient timing

Traditional nutrient timing guidelines are typically based on the needs of endurance athletes. For example, it is common lore that post-exercise carbohydrate must elicit a substantial glycemic and insulinemic response in order to optimize recovery. The origin of this recommendation can be traced back to 1988, when Ivy et al. [70] put fasted subjects through a glycogen-depleting cycling bout and compared the rate of glycogen resynthesis from a carbohydrate solution (2 g/kg) consumed either immediately after, or two hours after the bout. Glycogen storage was 2–3 times faster in the immediate condition during four hours post-exercise resulting in greater glycogen storage at four hours.

These findings initiated the faster-is-better post-exercise guideline for carbohydrate. However, complete glycogen resynthesis to pre-trained levels can occur well within 24 hours given sufficient total carbohydrate intake. Jentjens and Jeukendrup [71] suggest that a between-bout period of eight hours or less is grounds for maximally expediting glycogen resynthesis. Therefore, the urgency of glycogen resynthesis is almost an exclusive concern of endurance athletes with multiple glycogen-depleting events separated by only a few hours. Bodybuilders in contest preparation may exceed a single training bout per day (e.g., weight-training in the morning, cardio in the evening). However, bodybuilders do not have the same performance objectives as multi-stage endurance competition, where the same muscle groups are trained to exhaustion in a repeated manner within the same day. Furthermore, resistance training bouts are typically not glycogen-depleting. High-intensity (70-80% of 1 RM), moderate-volume (6–9 sets per muscle group) bouts have been seen to reduce glycogen stores by roughly 36-39% [72,73].

A more relevant question to bodybuilding may be whether protein and/or amino acid timing affect LBM maintenance. With little exception [74], acute studies have consistently shown that ingesting protein/essential amino acids and carbohydrate near or during the training bout can increase muscle protein synthesis (MPS) and suppress muscle protein breakdown [75-79]. However, there is a disparity between short- and long-term outcomes in studies examining the effect of nutrient timing on resistance training adaptations.

To-date, only a minority of chronic studies have shown that specific timing of nutrients relative to the resistance training bout can affect gains in muscular size and/or strength. Cribb and Hayes [80] found that timing a supplement consisting of 40 g protein, 43 g carbohydrate, and 7 g creatine immediately pre- and post-exercise resulted in greater size and strength gains than positioning the supplement doses away from the training bout. Additionally, Esmarck et al. [81] observed greater hypertrophy in subjects who ingested a supplement (10 g protein, 8 g carbohydrate, 3 g fat) immediately post-exercise than subjects who delayed the supplement 2 hours post-exercise.

In contrast, the majority of chronic studies have not supported the effectiveness of timing nutrients (protein in particular) closely around the training bout. Burk et al. [82], found that a *time-divided* regimen (two 35 g protein doses consumed at far-off points in the morning and evening away from the afternoon training bout) caused slightly better gains in squat strength and fat-free mass than the *time-focused* regimen, where the protein supplement doses were consumed in the morning, and then again immediately prior to the resistance training bout. Hoffman et al. [83] found no significant differences in strength gains or body composition when comparing an immediate pre- and post-exercise supplement ingestion (each dose provided 42 g protein) with the supplement ingested distantly separate from each side of the training bout. This lack of effect was attributed to the subjects’ sufficient daily protein consumption combined with their advanced lifting status. Wycherley et al. [84] examined the effects of varying nutrient timing on overweight and obese diabetics. A meal containing 21 g protein consumed immediately before resistance training was compared with its consumption at least two hours after training. No significant differences in weight loss, strength gain, or cardio metabolic risk factor reductions were seen. Most recently, Weisgarber et al. [85] observed no significant effect on muscle mass and strength from consuming whey protein immediately before or throughout resistance training.

It’s important to note that other chronic studies are referred to as nutrient timing studies, but have not matched total protein intake between conditions. These studies examined the effect of additional nutrient content, rather than examining the effect of different temporal placement of nutrients relative to the training bout. Thus, they cannot be considered true timing comparisons. Nevertheless, these studies have yielded inconsistent results. Willoughby et al. [86] found that 10 weeks of resistance training supplemented with 20 g protein and amino acids 1 hour pre- and post-exercise increased strength performance and MPS compared to an energy-matched carbohydrate placebo. Hulmi et al. [87] found that 21 weeks of supplementing 15 g of whey before and after resistance training increased size and altered gene expression favorably towards muscle anabolism in the vastus lateralis. In contrast to the previous 2 studies, Verdijk et al. [88] found no significant effect of 10 g protein timed immediately before and after resistance training over a 12-week period. The authors attributed this lack of effect to an adequate total daily protein intake. Recently, a 12-week trial by Erksine et al. [89] reported a lack of effect of 20 g protein taken pre- and post-exercise compared to placebo.

The disparity of outcomes between the acute and chronic studies could also potentially be due to a longer “anabolic window” than traditionally thought. Burd and colleagues [90] found that resistance training to failure can cause an increased anabolic response to protein feedings that can last up to 24 hours. Demonstrating the body's drive toward equilibrium, Deldicque et al. [91] observed a greater intramyocellular anabolic response in fasted compared to fed subjects given a post-exercise carbohydrate/protein/leucine mixture. This result suggests that the body is capable of anabolic supercompensation despite the inherently catabolic nature of fasted resistance training. These data, in addition to the previously discussed chronic studies, further support the idea that macronutrient totals by the end of the day may be more important than their temporal placement relative to the training bout.

There are additional factors that might explain the lack of consistent effectiveness of nutrient timing in chronic studies. Training status of the subjects could influence outcomes since novice trainees tend to respond similarly to a wider variety of stimuli. Another possible explanation for the lack of timing effects is the protein dose used, 10–20 g, which may not be sufficient to elicit a maximal anabolic response. MPS rates have been shown to plateau with a post-exercise dose of roughly 20 g of high-quality protein [92]. However, in subsequent research on older subjects, Yang et al. [93] observed that an even higher post-exercise protein dose (40 g) stimulated MPS to a greater extent than 10 g or 20 g.

In addition to the paucity of studies using ample protein doses, there is a lack of investigation of protein-carbohydrate combinations. Only Cribb and Hayes [80] have compared substantial doses of both protein (40 g) and carbohydrate (43 g) taken immediately surrounding, versus far apart from both sides of the training bout. Nearly double the lean mass gains were seen in the proximally timed compared to the distally timed condition. However, acute studies examining the post-exercise anabolic response elicited by co-ingesting carbohydrate with protein have thus far failed to show significant effects given a sufficient protein dose of approximately 20–25 g [94,95]. These results concur with previous data indicating that only moderate insulin elevations (15–30 mU/L) are required to maximize net muscle protein balance in the presence of elevated plasma amino acids [96]. Koopman et al. [97] observed a similar lack of carbohydrate-mediated anabolic effect when protein was administered at 0.3 g/kg/hr in the post-exercise recovery period.

**Figure 1:**
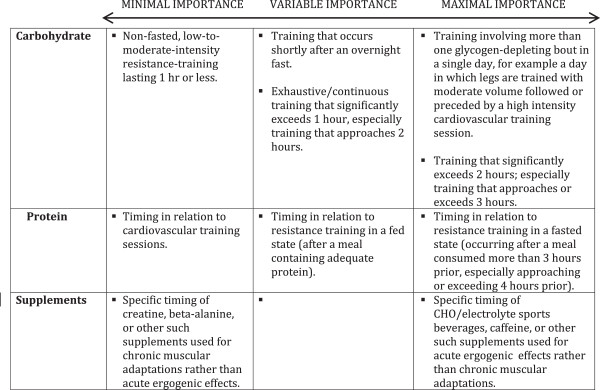
Continuum of nutrient & supplement timing importance.

Questions remain about the utility of consuming protein and/or carbohydrate during bodybuilding-oriented training bouts. Since these bouts typically do not resemble endurance bouts lasting 2 hours or more, nutrient consumption during training is not likely to yield any additional performance-enhancing or muscle -sparing benefits if proper pre-workout nutrition is in place. In the exceptional case of resistance training sessions that approach or exceed two hours of exhaustive, continuous work, it might be prudent to employ tactics that maximize endurance capacity while minimizing muscle damage. This would involve approximately 8–15 g protein co-ingested with 30–60 g carbohydrate in a 6-8% solution per hour of training [98]. Nutrient timing is an intriguing area of study that focuses on what might clinch the competitive edge. In terms of practical application to resistance training bouts of typical length, Aragon and Schoenfeld [99] recently suggested a protein dose corresponding with 0.4-0.5 g/kg bodyweight consumed at both the pre- and post-exercise periods. However, for objectives relevant to bodybuilding, the current evidence indicates that the global macronutrient composition of the diet is likely the most important nutritional variable related to chronic training adaptations. Figure 1 below provides a continuum of importance with bodybuilding-specific context for nutrient timing.

#### Meal frequency

Previous optimal meal frequency studies have lacked structured resistance training protocols. Moreover, there are no studies that specifically examined meal frequency in bodybuilders, let alone during contest preparation conditions. Despite this limitation, the available research has consistently refuted the popular belief that a grazing pattern (smaller, more frequent meals) raises energy expenditure compared to a gorging pattern (larger, less frequent meals). Disparate feeding patterns ranging from two to seven meals per day have been compared in tightly controlled studies using metabolic chambers, and no significant differences in 24-hour thermogenesis have been detected [100,101]. It should be noted that irregular feeding patterns across the week, as opposed to maintaining a stable daily frequency, has been shown to decrease post-prandial thermogenesis [102] and adversely affect insulin sensitivity and blood lipid profile [103]. However, relevance of the latter findings might be limited to sedentary populations, since regular exercise is well-established in its ability to improve insulin sensitivity and blood lipids.

Bodybuilders typically employ a higher meal frequency in an attempt to optimize fat loss and muscle preservation. However, the majority of chronic experimental studies have failed to show that different meal frequencies have different influences on bodyweight or body composition [104-108]. Of particular interest is the research examining the latter, since the preservation of muscle mass during fat loss is a paramount concern in the pre-contest phase. A recent review by Varady [109] examined 11 daily caloric restriction (CR) studies and 7 intermittent calorie restriction (ICR) studies. CR involved a linear consumption of 15-60% of baseline needs every day, while ICR alternated ad libitum ‘feed’ days with ‘fast’ days involving partial or total food intake restriction. It was concluded that although both types have similar effects on total bodyweight reduction, ICR has thus far been more effective for retaining lean mass. Three of the ICR studies showed no significant decrease in LBM, while all of the CR studies showed decreased LBM. However, the majority of the ICR trials used bioelectrical impedance analysis (BIA) to measure body composition, while the majority of CR studies used dual X-ray absorptiometry (DXA) or magnetic resonance imaging (MRI). These methods have been shown to have greater accuracy than BIA [110-112], so the results of Varady’s [109] analysis should be interpreted with caution. Along these lines, Stote et al. [113] found that compared to three meals per day, one meal per day caused slightly more weight and fat loss. Curiously, the one meal per day group also showed a slight gain in lean mass, but this could have been due to the inherent error in BIA for body composition assessment.

To-date, only two experimental studies have used trained, athletic subjects. Iwao et al. [114] found that boxers consuming six meals a day lost less LBM and showed lower molecular measures of muscle catabolism than the same diet consumed in two meals per day. However, limitations to this study included short trial duration, subpar assessment methods, a small sample size, and a 1200 kcal diet which was artificially low compared to what this population would typically carry out in the long-term. It is also important to note that protein intake, at 20% of total kcal, amounted to 60 g/day which translates to slightly under 1.0 g/kg. To illustrate the inadequacy of this dose, Mettler et al. [29] showed that protein as high as 2.3 g/kg and energy intake averaging 2022 kcal was still not enough to completely prevent LBM loss in athletes under hypocaloric conditions. The other experimental study using athletic subjects was by Benardot et al. [115], who compared the effects of adding three 250 kcal between-meal snacks with the addition of a noncaloric placebo. A significant increase in anaerobic power and lean mass was seen in the snacking group, with no such improvements seen in the placebo group. However, it is not possible to determine if the superior results were the result of an increased meal frequency or increased caloric intake.

A relatively recent concept with potential application to meal frequency is that a certain minimum dose of leucine is required in order to stimulate muscle protein synthesis. Norton and Wilson [116] suggested that this threshold dose is approximately 0.05 g/kg, or roughly 3 g leucine per meal to saturate the mTOR signaling pathway and trigger MPS. A related concept is that MPS can diminish, or become 'refractory' if amino acids are held at a constant elevation. Evidence of the refractory phenomenon was shown by Bohé et al. [117], who elevated plasma amino acid levels in humans and observed that MPS peaked at the 2-hour mark, and rapidly declined thereafter despite continually elevated blood amino acid levels. For the goal of maximizing the anabolic response, the potential application of these data would be to avoid spacing meals too closely together. In addition, an attempt would be made to reach the leucine threshold with each meal, which in practical terms would be to consume at least 30–40 g high-quality protein per meal. In relative agreement, a recent review by Phillips and Van Loon [28] recommends consuming one's daily protein requirement over the course of three to four isonitrogenous meals per day in order to maximize the acute anabolic response per meal, and thus the rate of muscle gain.

It is important to note that the leucine threshold and the refractory nature of MPS are not based on human feeding studies that measure concrete outcomes over the long-term. These ideas are largely based on mechanistic studies whose data was derived via steady intravenous infusion of amino acids [117,118]. Long-term studies are needed to determine if the refractory nature of MPS seen in acute infusion data would have any real impact on the gain or preservation of LBM at various meal frequencies.

Munster and Saris [119] recently shed further light on what might be optimal in the context of pre-contest dieting. Lean, healthy subjects underwent 36-hour periods in a respiration chamber. Interestingly, three meals per day resulted in higher protein oxidation and RMR, along with lower overall blood glucose concentrations than an isoenergetic diet composed of 14 meals per day. The lower glucose AUC observed in this study is in agreement with previous research by Holmstrup et al. [120], who reported lower 12-hour glucose concentrations as a result of consuming three high-carbohydrate meals compared to the equivalent distributed over the course of six meals. Another interesting finding by Munster and Saris [119] was lower hunger and higher satiety ratings in the lower meal frequency condition. This finding concurred with previous work by Leidy et al. [121], who compared varying protein levels consumed across either three or six meals per day. Predictably, the higher-protein level (25% vs. 14%) promoted greater satiety. Interestingly, the higher meal frequency led to lower daily fullness ratings regardless of protein level. Meal frequency had no significant impact on ghrelin levels, regardless of protein intake. PYY, a gut peptide associated with satiety, was 9% lower in the higher meal frequency condition. However, Arciero et al. [122] recently found that six meals per day in a high-protein condition (35% of total energy) were superior to three meals with a high-protein or traditional protein intake (15% of total energy) for improving body composition in overweight subjects. The discrepancy between Leidy et al’s short-term effects and Arciero’s chronic effects warrants further study, preferably in subjects undergoing progressive resistance training.

Other common meal frequencies (i.e., 4 or 5 meals per day) have eluded scientific investigation until very recently. Adechian et al. [123] compared whey versus casein consumed in either a 'pulse' meal pattern (8/80/4/8%) or a 'spread' pattern (25/25/25/25%) over a six week hypocaloric period. No significant changes were seen in body composition between conditions. These outcomes challenge Phillips and Van Loon's recommendation for protein-rich meals throughout the day to be isonitrogenous (40). Moore et al. [124] compared evenly spaced distributions of two, four, and eight meals consumed after a fasted, acute bout of bilateral knee extension. A trend toward a small and moderate increase in net protein balance was seen in the four meal and eight meal conditions, respectively, compared to the two meal condition. Subsequent work by Areta et al. [125] using the same dosing comparison found that the four meal treatment (20 g protein per meal) caused the greatest increase in myofibrillar protein synthesis. A limitation of both of the previous studies was the absence of other macronutrients (aside from protein in whey) consumed during the 12-hour postexercise period. This leaves open questions about how a real-world scenario with mixed meals might have altered the outcomes. Furthermore, these short-term responses lack corroboration in chronic trials measuring body composition and/or exercise performance outcomes.

The evidence collectively suggests that extreme lows or highs in meal frequency have the potential to threaten lean mass preservation and hunger control during bodybuilding contest preparation. However, the functional impact of differences in meal frequency at moderate ranges (e.g., 3–6 meals per day containing a minimum of 20 g protein each) are likely to be negligible in the context of a sound training program and properly targeted total daily macronutrition.

### Nutritional supplementation

When preparing for a bodybuilding contest, a competitor primarily focuses on resistance training, nutrition, and cardiovascular training; however, supplements may be used to further augment preparation. This section will discuss the scientific evidence behind several of the most commonly used supplements by bodybuilders. However, natural bodybuilding federations have extensive banned substance lists [126]; therefore, banned substances will be omitted from this discussion. It should be noted that there are considerably more supplements that are used by bodybuilders and sold on the market. However, an exhaustive review of all of the supplements commonly used by bodybuilders that often lack supporting data is beyond the scope of this paper. In addition, we have omitted discussion of protein supplements because they are predominantly used in the same way that whole food protein sources are used to reach macronutrient targets; however, interested readers are encouraged to reference the ISSN position stand on protein and exercise [127].

#### Creatine

Creatine monohydrate (CM) has been called the most ergogenic and safe supplement that is legally available [128]. Supplementation of healthy adults has not resulted in any reported adverse effects or changes in liver or kidney function [129]. Numerous studies have found significantly increased muscle size and strength when CM was added to a strength training program [130-134]. In many of these studies, 1-2 kg increases in total body mass were observed after CM loading of 20 g/day for 4–28 days [135]. However, the loading phase may not be necessary. Loading 20 g CM per day has been shown to increase muscle total creatine by approximately 20 percent and this level of muscle creatine was maintained with 2 g CM daily for 30 days [136]. However, the same study also observed a 20 percent increase in muscle creatine when 3 g CM was supplemented daily for 28 days, indicating the loading phase may not be necessary to increase muscle creatine concentrations.

Recently, alternative forms of creatine, such as creatine ethyl ester (CEE) and Kre Alkalyn (KA) have been marketed as superior forms of creatine to CM; however, as of this time these claims have not been supported by scientific studies. Tallon and Child [137,138] found that a greater portion of CEE and KA are degraded in the stomach than CM. Additionally, recent investigations have shown that 28–42 days of CEE or KA supplementation did not increase muscle creatine concentrations more than CM [139,140]. Thus, it appears that CM may be the most effective form of creatine.

#### Beta-alanine

Beta-alanine (BA) is becoming an increasingly popular supplement among bodybuilders. Once consumed, BA enters the circulation and is up-taken by skeletal muscle where it is used to synthesize carnosine, a pH buffer in muscle that is particularly important during anaerobic exercise such as sprinting or weightlifting [141]. Indeed, consumption of 6.4 g BA daily for four weeks has been shown to increase muscle carnosine levels by 64.2% [142]. Moreover, supplementation with BA for 4–10 weeks has been shown to increase knee extension torque by up to 6% [143], improve workload and time to fatigue during high intensity cardio [144-148], improve muscle resistance to fatigue during strength training [149], increase lean mass by approximately 1 kg [147] and significantly reduce perceptions of fatigue [150]. Additionally, the combination of BA and CM may increase performance of high intensity endurance exercise [151] and has been shown to increase lean mass and decrease body fat percentage more than CM alone [152]. However, not all studies have shown improvements in performance with BA supplementation [143,153,154]. To clarify these discrepancies, Hobson et al. [155] conducted a meta-analysis of 15 studies on BA supplementation and concluded that BA significantly increased exercise capacity and improved exercise performance on 60-240 s (ES = 0.665) and >240 s (ES = 0.368) exercise bouts.

Although BA appears to improve exercise performance, the long-term safety of BA has only been partially explored. Currently, the only known side effect of BA is unpleasant symptoms of parasthesia reported after consumption of large dosages; however, this can be minimized through consumption of smaller dosages throughout the day [142]. While BA appears to be relatively safe in the short-term, the long-term safety is unknown. In cats, an addition of 5 percent BA to drinking water for 20 weeks has been shown to deplete taurine and result in damage to the brain; however, taurine is an essential amino acid for cats but not for humans and it is unknown if the smaller dosages consumed by humans could result in similar effects [156]. BA may increase exercise performance and increase lean mass in bodybuilders and currently appears to be safe; however, studies are needed to determine the long-term safety of BA consumption.

#### HMB

Beta-hydroxy-beta-methylbutyrate (HMB) is a metabolite of the amino acid leucine that has been shown to decrease muscle protein catabolism and increase muscle protein synthesis [157,158]. The safety of HMB supplementation has been widely studied and no adverse effects on liver enzymes, kidney function, cholesterol, white blood cells, hemoglobin, or blood glucose have been observed [159-161]. Furthermore, two meta-analyses on HMB supplementation have concluded that HMB is safe and does not result in any major side effects [159,160]. HMB may actually decrease blood pressure, total and LDL cholesterol, especially in hypercholesterolemic individuals.

HMB is particularly effective in catabolic populations such as the elderly and patients with chronic disease [162]. However, studies on the effectiveness of HMB in trained, non-calorically restricted populations have been mixed. Reasons for discrepancies in the results of HMB supplementation studies in healthy populations may be due to many factors including clustering of data in these meta-analysis to include many studies from similar groups, poorly designed, non-periodized training protocols, small sample sizes, and lack of specificity between training and testing conditions [163]. However, as a whole HMB appears to be effective in a majority of studies with longer-duration, more intense, periodized training protocols and may be beneficial to bodybuilders, particularly during planned over-reaching phases of training [164]. While the authors hypothesize that HMB may be effective in periods of increased catabolism, such as during contest preparation, the efficacy of HMB on maintenance of lean mass in dieting athletes has not been investigated in a long-term study. Therefore, future studies are needed to determine the effectiveness of HMB during caloric restriction in healthy, lean, trained athletes.

#### Branched chain amino acids

Branched chain amino acids (BCAA’s) make up 14-18% of amino acids in skeletal muscle proteins and are quite possibly the most widely used supplements among natural bodybuilders [165]. Of the BCAA’s, leucine is of particular interest because it has been shown to stimulate protein synthesis to an equal extent as a mixture of all amino acids [166]. However, ingestion of leucine alone can lead to depletion of plasma valine and isoleucine; therefore, all three amino acids need to be consumed to prevent plasma depletion of any one of the BCAA’s [167]. Recently, the safe upper limit of leucine was set at 550 mg/kg bodyweight/day in adult men; however, future studies are needed to determine the safe upper limit for both other populations and a mixture of all 3 BCAA’s [168].

Numerous acute studies in animals and humans have shown that consumption of either essential amino acids, BCAA’s, or leucine either at rest or following exercise increases skeletal muscle protein synthesis, decreases muscle protein degradation, or both [27,169-172]; however, there are few long-term studies of BCAA supplementation in resistance-trained athletes. Stoppani et al. [173] supplemented trained subjects with either 14 g BCAAs, whey protein, or a carbohydrate placebo for eight weeks during a periodized strength training routine. After training the BCAA group had a 4 kg increase in lean mass, 2% decrease in body fat percentage, and 6 kg increase in bench press 10 repetition maximum. All changes were significant compared to the other groups. However, it should be noted that this data is only available as an abstract and has yet to undergo the rigors of peer-review.

The use of BCAA’s between meals may also be beneficial to keep protein synthesis elevated. Recent data from animal models suggest that consumption of BCAA’s between meals can overcome the refractory response in protein synthesis that occurs when plasma amino acids are elevated, yet protein synthesis is reduced [174]. However, long-term human studies examining the effects of a diet in which BCAA’s are consumed between meals on lean mass and strength have not been done to date. It should also be noted that BCAA metabolism in humans and rodents differ and the results from rodent studies with BCAA’s may not translate in human models [175]. Therefore, long-term studies are needed in humans to determine the effectiveness of this practice.

Based on the current evidence, it is clear BCAA’s stimulate protein synthesis acutely and one study [173] has indicated that BCAA’s may be able to increase lean mass and strength when added to a strength training routine; however, additional long-term studies are needed to determine the effects of BCAA’s on lean mass and strength in trained athletes. In addition, studies are needed on the effectiveness of BCAA supplementation in individuals following a vegetarian diet in which consumption of high-quality proteins are low as this may be population that may benefit from BCAA consumption. Furthermore, the effects of BCAA ingestion between meals needs to be further investigated in a long-term human study.

#### Arginine

“NO supplements” containing arginine are consumed by bodybuilders pre-workout in an attempt to increase blood flow to the muscle during exercise, increase protein synthesis, and improve exercise performance. However, there is little scientific evidence to back these claims. Fahs et al. [176] supplemented healthy young men with 7 g arginine or a placebo prior to exercise and observed no significant change in blood flow following exercise. Additionally, Tang et al. [177] supplemented either 10 g arginine or a placebo prior to exercise and found no significant increase in blood flow or protein synthesis following exercise. Moreover, arginine is a non essential amino acid and prior work has established that essential amino acids alone stimulate protein synthesis [178]. Based on these findings, it appears that arginine does not significantly increase blood flow or enhance protein synthesis following exercise.

The effects of arginine supplementation on performance are controversial. Approximately one-half of acute and chronic studies on arginine and exercise performance have found significant benefits with arginine supplementation, while the other one-half has found no significant benefits [179]. Moreover, Greer et al. [180] found that arginine supplementation significantly reduced muscular endurance by 2–4 repetitions on chin up and push up endurance tests. Based on these results, the authors of a recent review concluded that arginine supplementation had little impact on exercise performance in healthy individuals [181]. Although the effects of arginine on blood flow, protein synthesis, and exercise performance require further investigation, dosages commonly consumed by athletes are well below the observed safe level of 20 g/d and do not appear to be harmful [182].

#### Citrulline malate

Citrulline malate (CitM) has recently become a popular supplement among bodybuilders; however, there has been little scientific research in healthy humans with this compound. CitM is hypothesized to improve performance through three mechanisms: 1) citrulline is important part of the urea cycle and may participate in ammonia clearance, 2) malate is a tricarboxylic acid cycle intermediate that may reduce lactic acid accumulation, and 3) citrulline can be converted to arginine; however, as discussed previously, arginine does not appear to have an ergogenic effect in young healthy athletes so it is unlikely CitM exerts an ergogenic effect through this mechanism [179,183].

Supplementation with CitM for 15 days has been shown to increase ATP production by 34% during exercise, increase the rate of phosphocreatine recovery after exercise by 20%, and reduce perceptions of fatigue [184]. Moreover, ingestion of 8 g CitM prior to a chest workout significantly increased repetitions performed by approximately 53% and decreased soreness by 40% at 24 and 48 hours post-workout [183]. Furthermore, Stoppani et al. [173] in an abstract reported a 4 kg increase in lean mass, 2 kg decrease in body fat percentage, and a 6 kg increase in 10 repetition maximum bench press after consumption of a drink containing 14 g BCAA, glutamine, and CitM during workouts for eight weeks; although, it is not clear to what degree CitM contributed to the outcomes observed. However, not all studies have supported ergogenic effects of CitM. Sureda et al. [185] found no significant difference in race time when either 6 g CitM or a placebo were consumed prior to a 137 km cycling stage. Hickner et al. [186] found that treadmill time to exhaustion was significantly impaired, with the time taken to reach exhaustion occurring on average seven seconds earlier following CitM consumption.

Additionally, the long-term safety of CitM is unknown. Therefore, based on the current literature a decision on the efficacy of CitM cannot be made. Future studies are needed to conclusively determine if CitM is ergogenic and to determine its long term safety.

#### Glutamine

Glutamine is the most abundant non-essential amino acid in muscle and is commonly consumed as a nutritional supplement. Glutamine supplementation in quantities below 14 g/d appear to be safe in healthy adults [182]; however, at present there is little scientific evidence to support the use of glutamine in healthy athletes [187]. Acutely, glutamine supplementation has not been shown to significantly improve exercise performance [188,189], improve buffering capacity [189], help to maintain immune function or reduce muscle soreness after exercise [187]. Long-term supplementation studies including glutamine in cocktails along with CM, whey protein, BCAA’s, and/or CitM have shown 1.5 – 2 kg increases in lean mass and 6 kg increase in 10RM bench press strength [173,190]. However, the role of glutamine in these changes is unclear. Only one study [191] has investigated the effects of glutamine supplementation alone in conjunction with a six week strength training program. No significant differences in muscle size, strength, or muscle protein degradation were observed between groups. Although the previous studies do not support the use of glutamine in bodybuilders during contest preparation, it should be noted that glutamine may be beneficial for gastrointestinal health and peptide uptake in stressed populations [192]; therefore, it may be beneficial in dieting bodybuilders who represent a stressed population. As a whole, the results of previous studies do not support use of glutamine as an ergogenic supplement; however, future studies are needed to determine the role of glutamine on gastrointestinal health and peptide transport in dieting bodybuilders.

#### Caffeine

Caffeine is perhaps the most common pre-workout stimulant consumed by bodybuilders. Numerous studies support the use of caffeine to improve performance during endurance training [193,194], sprinting [195,196], and strength training [197-199]. However, not all studies support use of caffeine to improve performance in strength training [200,201]. It should be noted that many of the studies that found increases in strength training performance supplemented with larger (5–6 mg/kg) dosages of caffeine. However, this dosage of caffeine is at the end of dosages that are considered safe (6 mg/kg/day) [202]. Additionally, it appears that regular consumption of caffeine may result in a reduction of ergogenic effects [203]. Therefore, it appears that 5–6 mg/kg caffeine taken prior to exercise is effective in improving exercise performance; however, caffeine use may need to be cycled in order for athletes to obtain the maximum ergogenic effect.

#### Micronutrients

Several previous studies have observed deficiencies in intakes of micronutrients, such as vitamin D, calcium, zinc, magnesium, and iron, in dieting bodybuilders [3,17,18,204,205]. However, it should be noted that these studies were all published nearly 2 decades ago and that micronutrient deficiencies likely occurred due to elimination of foods or food groups and monotony of food selection [3,205]. Therefore, future studies are needed to determine if these deficiencies would present while eating a variety of foods and using the contest preparation approach described herein. Although the current prevalence of micronutrient deficiencies in competitive bodybuilders is unknown, based on the previous literature, a low-dose micronutrient supplement may be beneficial for natural bodybuilders during contest preparation; however, future studies are needed to verify this recommendation.

### Peak week

In an attempt to enhance muscle size and definition by reducing extracellular water content, many bodybuilders engage in fluid, electrolyte, and carbohydrate manipulation in the final days and hours before competing [2,60,206]. The effect of electrolyte manipulation and dehydration on visual appearance has not been studied, however it may be a dangerous practice [207]. Furthermore, dehydration could plausibly degrade appearance considering that extracellular water is not only present in the subcutaneous layer. A significant amount is located in the vascular system. Thus, the common practice of "pumping up" to increase muscle size and definition by increasing blood flow to the muscle with light, repetitive weight lifting prior to stepping on stage [208] could be compromised by dehydration or electrolyte imbalance. Furthermore, dehydration reduces total body hydration. A large percentage of muscle tissue mass is water and dehydration results in decreases in muscle water content [209] and therefore muscle size, which may negatively impact the appearance of muscularity.

In the final days before competing, bodybuilders commonly practice carbohydrate loading similar to endurance athletes in an attempt to raise muscle-glycogen levels and increase muscle size [4,18,60,208]. In the only direct study of this practice, no significant quantitative change in muscle girth was found to occur [208]. However, an isocaloric diet was used, with only a change in the percentage of carbohydrate contributing to the diet. If total calories had also been increased, greater levels of glycogen might have been stored which could have changed the outcome of this study. Additionally, unlike the subjects in this study bodybuilders prior to carbohydrate loading have reduced glycogen levels from a long calorically restricted diet and it is possible in this state that carbohydrate loading might effect a visual change. Furthermore, bodybuilding performance is measured subjectively, thus analysis of girth alone may not discern subtle visual changes which impact competitive success. Lastly, some bodybuilders alter the amount of carbohydrate loaded based on the visual outcome, increasing the amount if the desired visual change does not occur [60]. Thus, an analysis of a static carbohydrate load may not accurately represent the dynamic nature of actual carbohydrate loading practices.

In fact, in an observational study of competitive bodybuilders in the days before competition who loaded carbohydrates, subjects showed a 4.9% increase in biceps thickness the final day before competition compared to six weeks prior [4]. Although it is unknown if this was caused by increased muscle glycogen, it is unlikely it was due to muscle mass accrual since the final weeks of preparation are often marked by decreases not increases in LBM [6]. Future studies of this practice should include a qualitative analysis of visual changes and analyze the effects of concurrent increases in percentage of carbohydrates as well as total calories.

At this time it is unknown whether dehydration or electrolyte manipulation improves physique appearance. What is known is that these practices are dangerous and have the potential to worsen it. It is unclear if carbohydrate loading has an impact on appearance and if so, how significant the effect is. However, the recommended muscle-sparing practice by some researchers to increase the carbohydrate content of the diet in the final weeks of preparation [6] might achieve any proposed theoretical benefits of carbohydrate loading. If carbohydrate loading is utilized, a trial run before competition once the competitor has reached or nearly reached competition leanness should be attempted to develop an individualized strategy. However, a week spent on a trial run consuming increased carbohydrates and calories may slow fat loss, thus ample time in the diet would be required.

### Psychosocial issues

Competitive bodybuilding requires cyclical periods of weight gain and weight loss for competition. In a study by Anderson et al. [207], it was found that 46% of a group of male drug free bodybuilders reported episodes of binge eating after competitions. One third to half reported anxiety, short tempers or anger when preparing for competition and most (81.5%) reported preoccupation with food.

Competitive male bodybuilders exhibit high rates of weight and shape preoccupation, binge eating and bulimia nervosa. However, they exhibit less eating-related and general psychopathology compared to men already diagnosed with bulimia nervosa [210]. Often they are more focused on muscle gain versus fat loss when compared to males with eating disorders [211]. That being said, this may change during preparation for competition when body builders need to reduce body fat levels.

Muscle dysmorphia is higher in male competitive natural bodybuilders than in collegiate football players and non-competitive weight trainers for physique [212]. However, the psychosocial profile of competitive bodybuilders is rather complex. Despite exhibiting greater risk for eating disturbances and a greater psychological investment in their physical appearance, they may have greater levels of physique satisfaction compared to non-competitive weight lifters and athletically active men [213]. Also, male bodybuilders are not a body-image homogenous group when experience is taken into account. Novice bodybuilders show greater levels of dissatisfaction with their muscle size and greater tendencies towards unhealthy and obsessive behavior [214]. Furthermore, the physical effects of semi-starvation in men can approximate the signs and symptoms of eating disorders such as anorexia nervosa and bulimia nervosa [11]. Thus, many of the psychosocial effects and behaviors seen in competitive bodybuilders may be at least partially the result of a prolonged diet and becoming very lean. When these factors are all considered it may indicate that at least in men, competitive bodybuilding drives certain psychosocial behaviors, in addition to those with prior existing behaviors being drawn to the sport.

However this may not be as much the case with female bodybuilders. Walberg [215] when comparing competitive bodybuilders to non-competitive female weight lifters, found that among bodybuilders 42% used to be anorexic, 67% were terrified of becoming fat, and 50% experienced uncontrollable urges to eat. All of these markers were significantly higher in bodybuilders than in non-competitors. Furthermore, it was found that menstrual dysfunction was more common among the bodybuilders. In agreement with this finding, Kleiner et al. [2] reported that 25% of female bodybuilding competitors reported abnormal menstrual cycles.

Competitive bodybuilders are not alone in their risk and disposition towards behaviors that carry health concerns. Elite athletes in aesthetic and weight-class sports as a whole share these risks [216]. In some sports, minimum body fat percentages can be established and minimum hydration levels for weighing in can be set. However, because bodybuilding performance is directly impacted by body fat percentage and not by weight per se, these regulatory changes to the sport are unlikely. Therefore, competitors and trainers should be aware of the potential psychosocial risks involved with competition. Open and frequent communication on these topics should be practiced and competitors and trainers should be aware of the signs and symptoms of unhealthy behaviors. Early therapeutic intervention by specialists with experience in competitive bodybuilding and eating disorders should occur if disordered eating patterns or psychological distress occurs.

### Limitations

The primary limitation of this review is the lack of large-scale long-term studies on competitive natural bodybuilders. To circumvent this, long-term studies on skeletal muscle hypertrophy and body fat loss in athletic dieting human populations were preferentially selected. In the absence of such studies, acute studies and/or animal studies were selected.

## Competing interests

The authors declare that they have no competing interests.

## Authors’ contributions

ERH developed the concept for this manuscript and wrote the sections on caloric intake, macronutrients, psychosocial issues and “peak week”. AAA wrote the sections on nutrient timing and meal frequency. PJF wrote the abstract, methods, limitations, and the section on dietary supplementation. All authors read and approved the final manuscript.

## References

[B1] Scott BR Lockie RG Knight TJ Clark AC De Jonge XAKJ A comparison of methods to quantify the in-season training load of professional soccer players Int J Sports Physiol Perform 2013 8 195 202 23428492 10.1123/ijspp.8.2.195

[B2] Kleiner SM Bazzarre TL Litchford MD Metabolic profiles, diet, and health practices of championship male and female bodybuilders J Am Diet Assoc 1990 90 962 967 2365938

[B3] Sandoval WM Heyward VH Food selection patterns of bodybuilders Int J Sport Nutr 1991 1 61 68 1844403 10.1123/ijsn.1.1.61

[B4] Bamman MM Hunter GR Newton LE Roney RK Khaled MA Changes in body composition, diet, and strength of bodybuilders during the 12 weeks prior to competition J Sports Med Phys Fitness 1993 33 383 391 8035587

[B5] Lambert CP Frank LL Evans WJ Macronutrient considerations for the sport of bodybuilding Sports Med 2004 34 317 327 10.2165/00007256-200434050-00004 15107010

[B6] Maestu J Eliakim A Jurimae J Valter I Jurimae T Anabolic and catabolic hormones and energy balance of the male bodybuilders during the preparation for the competition J Strength Cond Res 2010 24 1074 1081 10.1519/JSC.0b013e3181cb6fd3 20300017

[B7] Hall KD What is the required energy deficit per unit weight loss? Int J Obes 2007 32 573 576 10.1038/sj.ijo.0803720 PMC2376744 17848938

[B8] MacLean PS Bergouignan A Cornier M-A Jackman MR Biology's response to dieting: the impetus for weight regain Am J Physiol Regul Integr Comp Physiol 2011 301 R581 R600 10.1152/ajpregu.00755.2010 21677272 PMC3174765

[B9] Camps SG Verhoef SP Westerterp KR Weight loss, weight maintenance, and adaptive thermogenesis Am J Clin Nutr 2013 97 990 994 10.3945/ajcn.112.050310 23535105

[B10] Johannsen DL Knuth ND Huizenga R Rood JC Ravussin E Hall KD Metabolic slowing with massive weight loss despite preservation of fat-free mass J Clin Endocrinol Metab 2012 97 2489 2496 10.1210/jc.2012-1444 22535969 PMC3387402

[B11] Keys A University of Minnesota. Laboratory of Physiological Hygiene The Biology Of Human Starvation 1950 Minneapolis: University of Minnesota Press

[B12] Trexler E Smith-Ryan A Norton L Metabolic adaptation to weight loss: implications for the athlete J Int Soc Sport Nutr 2014 11 7 10.1186/1550-2783-11-7 PMC3943438 24571926

[B13] Garthe I Raastad T Refsnes PE Koivisto A Sundgot-Borgen J Effect of two different weight-loss rates on body composition and strength and power-related performance in elite athletes Int J Sport Nutr Exerc Metab 2011 21 97 104 21558571 10.1123/ijsnem.21.2.97

[B14] Forbes GB Body fat content influences the body composition response to nutrition and exercise Ann N Y Acad Sci 2000 904 359 365 10865771 10.1111/j.1749-6632.2000.tb06482.x

[B15] Hall KD Body fat and fat-free mass inter-relationships: Forbes's theory revisited Br J Nutr 2007 97 1059 1063 10.1017/S0007114507691946 17367567 PMC2376748

[B16] Mero AA Huovinen H Matintupa O Hulmi JJ Puurtinen R Hohtari H Karila T Moderate energy restriction with high protein diet results in healthier outcome in women J Int Soc Sports Nutr 2010 7 4 10.1186/1550-2783-7-4 20205751 PMC2822830

[B17] Sandoval WM Heyward VH Lyons TM Comparison of body composition, exercise and nutritional profiles of female and male body builders at competition J Sports Med Phys Fitness 1989 29 63 70 2770270

[B18] Walberg-Rankin J Edmonds CE Gwazdauskas FC Diet and weight changes of female bodybuilders before and after competition Int J Sport Nutr 1993 3 87 102 8499941 10.1123/ijsn.3.1.87

[B19] Withers RT Noell CJ Whittingham NO Chatterton BE Schultz CG Keeves JP Body composition changes in elite male bodybuilders during preparation for competition Aust J Sci Med Sport 1997 29 11 16 9127683

[B20] van der Ploeg GE Brooks AG Withers RT Dollman J Leaney F Chatterton BE Body composition changes in female bodybuilders during preparation for competition Eur J Clin Nutr 2001 55 268 277 10.1038/sj.ejcn.1601154 11360131

[B21] Newton LE Hunter GR Bammon M Roney RK Changes in psychological state and self-reported diet during various phases of training in competitive bodybuilders J Strength Cond Res 1993 7 153 158

[B22] Butterfield GE Whole-body protein utilization in humans Med Sci Sports Exerc 1987 19 S157 S165 3316915

[B23] Lemon PW Beyond the zone: protein needs of active individuals J Am Coll Nutr 2000 19 513S 521S 10.1080/07315724.2000.10718974 11023001

[B24] Phillips SM Dietary protein for athletes: from requirements to metabolic advantage Appl Physiol Nutr Metab 2006 31 647 654 10.1139/h06-035 17213878

[B25] Phillips SM Moore DR Tang JE A critical examination of dietary protein requirements, benefits, and excesses in athletes Int J Sport Nutr Exerc Metab 2007 17 Suppl S58 S76 18577776 10.1123/ijsnem.17.s1.s58

[B26] Slater G Phillips SM Nutrition guidelines for strength sports: sprinting, weightlifting, throwing events, and bodybuilding J Sports Sci 2011 29 S67 S77 10.1080/02640414.2011.574722 21660839

[B27] Tipton KD Wolfe RR Protein and amino acids for athletes J Sports Sci 2004 22 65 79 10.1080/0264041031000140554 14971434

[B28] Phillips SM Van Loon LJ Dietary protein for athletes: from requirements to optimum adaptation J Sports Sci 2011 29 Suppl 1 S29 S38 22150425 10.1080/02640414.2011.619204

[B29] Mettler S Mitchell N Tipton KD Increased protein intake reduces lean body mass loss during weight loss in athletes Med Sci Sports Exerc 2010 42 326 337 19927027 10.1249/MSS.0b013e3181b2ef8e

[B30] Millward DJ Macronutrient intakes as determinants of dietary protein and amino acid adequacy J Nutr 2004 134 1588S 1596S 15173435 10.1093/jn/134.6.1588S

[B31] Stiegler P Cunliffe A The role of diet and exercise for the maintenance of fat-free mass and resting metabolic rate during weight loss Sports Med 2006 36 239 262 10.2165/00007256-200636030-00005 16526835

[B32] Walberg JL Leidy MK Sturgill DJ Hinkle DE Ritchey SJ Sebolt DR Macronutrient content of a hypoenergy diet affects nitrogen retention and muscle function in weight lifters Int J Sports Med 1988 9 261 266 10.1055/s-2007-1025018 3182156

[B33] Helms ER Zinn C Rowlands DS Brown SR A systematic review of dietary protein during caloric restriction in resistance trained lean athletes: a case for higher intakes Int J Sport Nutr Exerc Metab 2013 Epub ahead of print 10.1123/ijsnem.2013-0054 24092765

[B34] Elia M Stubbs RJ Henry CJ Differences in fat, carbohydrate, and protein metabolism between lean and obese subjects undergoing total starvation Obes Res 1999 7 597 604 10.1002/j.1550-8528.1999.tb00720.x 10574520

[B35] Phillips SM Protein requirements and supplementation in strength sports Nutrition 2004 20 689 695 10.1016/j.nut.2004.04.009 15212752

[B36] Tarnopolsky MA Building muscle: nutrition to maximize bulk and strength adaptations to resistance exercise training Eur J Sport Sci 2008 8 67 76 10.1080/17461390801919128

[B37] Tipton KD Protein for adaptations to exercise training Eur J Sport Sci 2008 8 107 118 10.1080/17461390801919102

[B38] Wilson J Wilson GJ Contemporary issues in protein requirements and consumption for resistance trained athletes J Int Soc Sports Nutr 2006 3 7 27 10.1186/1550-2783-3-1-7 18500966 PMC2129150

[B39] Celejowa I Homa M Food intake, nitrogen and energy balance in Polish weight lifters, during a training camp Nutr Metab 1970 12 259 274 10.1159/000175300 5510411

[B40] Pasiakos SM Cao JJ Margolis LM Sauter ER Whigham LD McClung JP Rood JC Carbone JW Combs GF Jr Young AJ Effects of high-protein diets on fat-free mass and muscle protein synthesis following weight loss: a randomized controlled trial FASEB J 2013 27 3837 3847 10.1096/fj.13-230227 23739654

[B41] Leveritt M Abernethy PJ Effects of carbohydrate restriction on strength performance J Strength Cond Res 1999 13 52 57

[B42] Haff GG Koch AJ Potteiger JA Kuphal KE Magee LM Green SB Jakicic JJ Carbohydrate supplementation attenuates muscle glycogen loss during acute bouts of resistance exercise Int J Sport Nutr Exerc Metab 2000 10 326 339 10997956 10.1123/ijsnem.10.3.326

[B43] MacDougall JD Ray S Sale DG McCartney N Lee P Garner S Muscle substrate utilization and lactate production Can J Appl Physiol 1999 24 209 215 10.1139/h99-017 10364416

[B44] Layman DK Boileau RA Erickson DJ Painter JE Shiue H Sather C Christou DD A reduced ratio of dietary carbohydrate to protein improves body composition and blood lipid profiles during weight loss in adult women J Nutr 2003 133 411 417 12566476 10.1093/jn/133.2.411

[B45] Layman DK Baum JI Dietary protein impact on glycemic control during weight loss J Nutr 2004 134 968S 973S 15051856 10.1093/jn/134.4.968S

[B46] Halton TL Hu FB The effects of high protein diets on thermogenesis, satiety and weight loss: a critical review J Am Coll Nutr 2004 23 373 385 10.1080/07315724.2004.10719381 15466943

[B47] Veldhorst M Smeets A Soenen S Hochstenbach-Waelen A Hursel R Diepvens K Lejeune M Luscombe-Marsh N Westerterp-Plantenga M Protein-induced satiety: effects and mechanisms of different proteins Physiol Behav 2008 94 300 307 10.1016/j.physbeh.2008.01.003 18282589

[B48] Westerterp-Plantenga MS Protein intake and energy balance Regul Pept 2008 149 67 69 10.1016/j.regpep.2007.08.026 18448177

[B49] Smeets AJ Soenen S Luscombe-Marsh ND Ueland O Westerterp-Plantenga MS Energy expenditure, satiety, and plasma ghrelin, glucagon-like peptide 1, and peptide tyrosine-tyrosine concentrations following a single high-protein lunch J Nutr 2008 138 698 702 18356323 10.1093/jn/138.4.698

[B50] Cook CM Haub MD Low-carbohydrate diets and performance Curr Sports Med Rep 2007 6 225 229 17617997

[B51] Volek JS Kraemer WJ Bush JA Incledon T Boetes M Testosterone and cortisol in relationship to dietary nutrients and resistance exercise J Appl Physiol 1997 82 49 54 10.1063/1.365847 9029197

[B52] Sallinen J Pakarinen A Ahtiainen J Kraemer WJ Volek JS Häkkinen K Relationship between diet and serum anabolic hormone responses to heavy-resistance exercise in men Int J Sports Med 2004 25 627 633 10.1055/s-2004-815818 15532008

[B53] Hämäläinen EK Adlercreutz H Puska P Pietinen P Decrease of serum total and free testosterone during a low-fat high-fibre diet J Steroid Biochem 1983 18 369 370 10.1016/0022-4731(83)90117-6 6298507

[B54] Dorgan JF Judd JT Longcope C Brown C Schatzkin A Clevidence BA Campbell WS Nair PP Franz C Kahle L Taylor PR Effects of dietary fat and fiber on plasma and urine androgens and estrogens in men: a controlled feeding study Am J Clin Nutr 1996 64 850 855 8942407 10.1093/ajcn/64.6.850

[B55] Hämäläinen EK Adlercreutz H Puska P Pietinen P Diet and serum sex hormones in healthy men J Steroid Biochem 1984 20 459 464 10.1016/0022-4731(84)90254-1 6538617

[B56] Suryanarayana BV Kent JR Meister L Parlow AF Pituitary-gonadal axis during prolonged total starvation in obese men Am J Clin Nutr 1969 22 767 770 5789477 10.1093/ajcn/22.6.767

[B57] Rossow LM Fukuda DH Fahs CA Loenneke JP Stout JR Natural bodybuilding competition preparation and recovery: a 12-month case study Int J Sports Physiol Perform 2013 8 582 592 23412685 10.1123/ijspp.8.5.582

[B58] Loucks AB Verdun M Heath EM Low energy availability, not stress of exercise, alters LH pulsatility in exercising women J Appl Physiol 1998 84 37 46 9451615 10.1152/jappl.1998.84.1.37

[B59] Bird SP Strength nutrition: maximizing your anabolic potential Strength Cond J 2010 32 80 86 10.1519/SSC.0b013e3181d5284e

[B60] Shephard RJ Electrolyte manipulation in female body-builders Br J Sports Med 1994 28 60 61 8044499 10.1136/bjsm.28.1.60-a PMC1332163

[B61] Too D Wakayama EJ Locati LL Landwer GE Effect of a precompetition bodybuilding diet and training regimen on body composition and blood chemistry J Sports Med Phys Fitness 1998 38 245 252 9830833

[B62] Sawyer JC Wood RJ Davidson PW Collins SM Matthews TD Gregory SM Paolone VJ Effects of a short-term carbohydrate-restricted diet on strength and power performance J Strength Cond Res 2013 27 2255 2262 10.1519/JSC.0b013e31827da314 23774282

[B63] Soenen S Bonomi AG Lemmens SGT Scholte J Thijssen MAMA van Berkum F Westerterp-Plantenga MS Relatively high-protein or ‘low-carb’ energy-restricted diets for body weight loss and body weight maintenance? Physiol Behav 2012 107 374 380 10.1016/j.physbeh.2012.08.004 22935440

[B64] Paoli A Grimaldi K D'Agostino D Cenci L Moro T Bianco A Palma A Ketogenic diet does not affect strength performance in elite artistic gymnasts J Int Soc Sports Nutr 2012 9 34 10.1186/1550-2783-9-34 22835211 PMC3411406

[B65] Essen-Gustavsson B Tesch PA Glycogen and triglyceride utilization in relation to muscle metabolic characteristics in men performing heavy-resistance exercise Eur J Appl Physiol 1990 61 5 10 10.1007/BF00236686 2289498

[B66] Goedecke JH Gibson ASC Grobler L Collins M Noakes TD Lambert EV Determinants of the variability in respiratory exchange ratio at rest and during exercise in trained athletes Am J Physiol Endocrinol Metab 2000 279 E1325 E1334 11093921 10.1152/ajpendo.2000.279.6.E1325

[B67] Cornier MA Donahoo WT Pereira R Gurevich I Westergren R Enerback S Eckel PJ Goalstone ML Hill JO Eckel RH Draznin B Insulin sensitivity determines the effectiveness of dietary macronutrient composition on weight loss in obese women Obes Res 2005 13 703 709 10.1038/oby.2005.79 15897479

[B68] Pendergast DR Leddy JJ Venkatraman JT A perspective on fat intake in athletes J Am Coll Nutr 2000 19 345 350 10.1080/07315724.2000.10718930 10872896

[B69] Turocy PS DePalma BF Horswill CA Laquale KM Martin TJ Perry AC Somova MJ Utter AC National athletic trainers' association position statement: safe weight loss and maintenance practices in sport and exercise J Athl Train 2011 46 322 336 21669104 10.4085/1062-6050-46.3.322 PMC3419563

[B70] Ivy JL Katz AL Cutler CL Sherman WM Coyle EF Muscle glycogen synthesis after exercise: effect of time of carbohydrate ingestion J Appl Physiol 1988 64 1480 1485 3132449 10.1152/jappl.1988.64.4.1480

[B71] Jentjens R Jeukendrup A Determinants of post-exercise glycogen synthesis during short-term recovery Sports Med 2003 33 117 144 10.2165/00007256-200333020-00004 12617691

[B72] Robergs RA Pearson DR Costill DL Fink WJ Pascoe DD Benedict MA Lambert CP Zachweija JJ Muscle glycogenolysis during differing intensities of weight-resistance exercise J Appl Physiol 1991 70 1700 1706 2055849 10.1152/jappl.1991.70.4.1700

[B73] Roy BD Tarnopolsky MA Influence of differing macronutrient intakes on muscle glycogen resynthesis after resistance exercise J Appl Physiol 1998 84 890 896 9480948 10.1152/jappl.1998.84.3.890

[B74] Fujita S Dreyer HC Drummond MJ Glynn EL Volpi E Rasmussen BB Essential amino acid and carbohydrate ingestion before resistance exercise does not enhance postexercise muscle protein synthesis J Appl Physiol 2009 106 1730 1739 10.1152/japplphysiol.90395.2008 18535123 PMC2681328

[B75] Baty JJ Hwang H Ding Z Bernard JR Wang B Kwon B Ivy JL The effect of a carbohydrate and protein supplement on resistance exercise performance, hormonal response, and muscle damage J Strength Cond Res 2007 21 321 329 17530986 10.1519/R-21706.1

[B76] Tipton KD Elliott TA Cree MG Aarsland AA Sanford AP Wolfe RR Stimulation of net muscle protein synthesis by whey protein ingestion before and after exercise Am J Physiol Endocrinol Metab 2007 292 E71 E76 16896166 10.1152/ajpendo.00166.2006

[B77] Bird SP Tarpenning KM Marino FE Liquid carbohydrate/essential amino acid ingestion during a short-term bout of resistance exercise suppresses myofibrillar protein degradation Metabolism 2006 55 570 577 10.1016/j.metabol.2005.11.011 16631431

[B78] Levenhagen DK Gresham JD Carlson MG Maron DJ Borel MJ Flakoll PJ Postexercise nutrient intake timing in humans is critical to recovery of leg glucose and protein homeostasis Am J Physiol Endocrinol Metab 2001 280 E982 E993 11350780 10.1152/ajpendo.2001.280.6.E982

[B79] Tipton KD Rasmussen BB Miller SL Wolf SE Owens-Stovall SK Petrini BE Wolfe RR Timing of amino acid-carbohydrate ingestion alters anabolic response of muscle to resistance exercise Am J Physiol Endocrinol Metab 2001 281 E197 E206 11440894 10.1152/ajpendo.2001.281.2.E197

[B80] Cribb PJ Hayes A Effects of supplement timing and resistance exercise on skeletal muscle hypertrophy Med Sci Sports Exerc 2006 38 1918 1925 10.1249/01.mss.0000233790.08788.3e 17095924

[B81] Esmarck B Andersen JL Olsen S Richter EA Mizuno M Kjaer M Timing of postexercise protein intake is important for muscle hypertrophy with resistance training in elderly humans J Physiol 2001 535 301 311 10.1111/j.1469-7793.2001.00301.x 11507179 PMC2278776

[B82] Burk A Timpmann S Medijainen L Vahi M Oopik V Time-divided ingestion pattern of casein-based protein supplement stimulates an increase in fat-free body mass during resistance training in young untrained men Nutr Res 2009 29 405 413 10.1016/j.nutres.2009.03.008 19628107

[B83] Hoffman JR Ratamess NA Tranchina CP Rashti SL Kang J Faigenbaum AD Effect of protein-supplement timing on strength, power, and body-composition changes in resistance-trained men Int J Sport Nutr Exerc Metab 2009 19 172 185 19478342 10.1123/ijsnem.19.2.172

[B84] Wycherley TP Noakes M Clifton PM Cleanthous X Keogh JB Brinkworth GD Timing of protein ingestion relative to resistance exercise training does not influence body composition, energy expenditure, glycaemic control or cardiometabolic risk factors in a hypocaloric, high protein diet in patients with type 2 diabetes Diabetes Obes Metab 2010 12 1097 1105 10.1111/j.1463-1326.2010.01307.x 20977582

[B85] Weisgarber KD Candow DG Vogt ES Whey protein before and during resistance exercise has no effect on muscle mass and strength in untrained young adults Int J Sport Nutr Exerc Metab 2012 22 463 469 22806076 10.1123/ijsnem.22.6.463

[B86] Willoughby DS Stout JR Wilborn CD Effects of resistance training and protein plus amino acid supplementation on muscle anabolism, mass, and strength Amino Acids 2007 32 467 477 10.1007/s00726-006-0398-7 16988909

[B87] Hulmi JJ Kovanen V Selanne H Kraemer WJ Hakkinen K Mero AA Acute and long-term effects of resistance exercise with or without protein ingestion on muscle hypertrophy and gene expression Amino Acids 2009 37 297 308 10.1007/s00726-008-0150-6 18661258

[B88] Verdijk LB Jonkers RA Gleeson BG Beelen M Meijer K Savelberg HH Wodzig WK Dendale P van Loon LJ Protein supplementation before and after exercise does not further augment skeletal muscle hypertrophy after resistance training in elderly men Am J Clin Nutr 2009 89 608 616 10.3945/ajcn.2008.26626 19106243

[B89] Erskine RM Fletcher G Hanson B Folland JP Whey protein does not enhance the adaptations to elbow flexor resistance training Med Sci Sports Exerc 2012 44 1791 1800 10.1249/MSS.0b013e318256c48d 22460474

[B90] Burd NA West DW Moore DR Atherton PJ Staples AW Prior T Tang JE Rennie MJ Baker SK Phillips SM Enhanced amino acid sensitivity of myofibrillar protein synthesis persists for up to 24 h after resistance exercise in young men J Nutr 2011 141 568 573 10.3945/jn.110.135038 21289204

[B91] Deldicque L De Bock K Maris M Ramaekers M Nielens H Francaux M Hespel P Increased p70s6k phosphorylation during intake of a protein-carbohydrate drink following resistance exercise in the fasted state Eur J Appl Physiol 2010 108 791 800 10.1007/s00421-009-1289-x 20187284

[B92] Moore DR Robinson MJ Fry JL Tang JE Glover EI Wilkinson SB Prior T Tarnopolsky MA Phillips SM Ingested protein dose response of muscle and albumin protein synthesis after resistance exercise in young men Am J Clin Nutr 2009 89 161 168 19056590 10.3945/ajcn.2008.26401

[B93] Yang Y Breen L Burd NA Hector AJ Churchward-Venne TA Josse AR Tarnopolsky MA Phillips SM Resistance exercise enhances myofibrillar protein synthesis with graded intakes of whey protein in older men Br J Nutr 2012 108 1 9 10.1017/S0007114511005204 22313809

[B94] Hamer HM Wall BT Kiskini A de Lange A Groen BB Bakker JA Gijsen AP Verdijk LB van Loon LJ Carbohydrate co-ingestion with protein does not further augment post-prandial muscle protein accretion in older men Nutr Metab (Lond) 2013 10 15 10.1186/1743-7075-10-15 23351781 PMC3585863

[B95] Staples AW Burd NA West DW Currie KD Atherton PJ Moore DR Rennie MJ Macdonald MJ Baker SK Phillips SM Carbohydrate does not augment exercise-induced protein accretion versus protein alone Med Sci Sports Exerc 2011 43 1154 1161 10.1249/MSS.0b013e31820751cb 21131864

[B96] Greenhaff PL Karagounis LG Peirce N Simpson EJ Hazell M Layfield R Wackerhage H Smith K Atherton P Selby A Rennie MJ Disassociation between the effects of amino acids and insulin on signaling, ubiquitin ligases, and protein turnover in human muscle Am J Physiol Endocrinol Metab 2008 295 E595 E604 10.1152/ajpendo.90411.2008 18577697 PMC2536736

[B97] Koopman R Beelen M Stellingwerff T Pennings B Saris WH Kies AK Kuipers H van Loon LJ Coingestion of carbohydrate with protein does not further augment postexercise muscle protein synthesis Am J Physiol Endocrinol Metab 2007 293 E833 E842 10.1152/ajpendo.00135.2007 17609259

[B98] Kerksick C Harvey T Stout J Campbell B Wilborn C Kreider R Kalman D Ziegenfuss T Lopez H Landis J Ivy JL Antonio J International Society of Sports Nutrition position stand: nutrient timing J Int Soc Sports Nutr 2008 5 17 10.1186/1550-2783-5-17 18834505 PMC2575187

[B99] Aragon AA Schoenfeld BJ Nutrient timing revisited: is there a post-exercise anabolic window? J Int Soc Sports Nutr 2013 10 5 10.1186/1550-2783-10-5 23360586 PMC3577439

[B100] Taylor MA Garrow JS Compared with nibbling, neither gorging nor a morning fast affect short-term energy balance in obese patients in a chamber calorimeter Int J Obes Relat Metab Disord 2001 25 519 528 10.1038/sj.ijo.0801572 11319656

[B101] de Venne WP V-v Westerterp KR Influence of the feeding frequency on nutrient utilization in man: consequences for energy metabolism Eur J Clin Nutr 1991 45 161 169 1905998

[B102] Farshchi HR Taylor MA Macdonald IA Decreased thermic effect of food after an irregular compared with a regular meal pattern in healthy lean women Int J Obes Relat Metab Disord 2004 28 653 660 10.1038/sj.ijo.0802616 15085170

[B103] Farshchi HR Taylor MA Macdonald IA Regular meal frequency creates more appropriate insulin sensitivity and lipid profiles compared with irregular meal frequency in healthy lean women Eur J Clin Nutr 2004 58 1071 1077 10.1038/sj.ejcn.1601935 15220950

[B104] Harvie MN Pegington M Mattson MP Frystyk J Dillon B Evans G Cuzick J Jebb SA Martin B Cutler RG Son TG Maudsley S Carlson OD Egan JM Flyvbjerg A Howell A The effects of intermittent or continuous energy restriction on weight loss and metabolic disease risk markers: a randomized trial in young overweight women Int J Obes 2011 35 714 727 10.1038/ijo.2010.171 PMC3017674 20921964

[B105] Soeters MR Lammers NM Dubbelhuis PF Ackermans M Jonkers-Schuitema CF Fliers E Sauerwein HP Aerts JM Serlie MJ Intermittent fasting does not affect whole-body glucose, lipid, or protein metabolism Am J Clin Nutr 2009 90 1244 1251 10.3945/ajcn.2008.27327 19776143

[B106] Arnal MA Mosoni L Boirie Y Houlier ML Morin L Verdier E Ritz P Antoine JM Prugnaud J Beaufrere B Mirand PP Protein feeding pattern does not affect protein retention in young women J Nutr 2000 130 1700 1704 10867039 10.1093/jn/130.7.1700

[B107] Arnal MA Mosoni L Boirie Y Houlier ML Morin L Verdier E Ritz P Antoine JM Prugnaud J Beaufrere B Mirand PP Protein pulse feeding improves protein retention in elderly women Am J Clin Nutr 1999 69 1202 1208 10357740 10.1093/ajcn/69.6.1202

[B108] La Bounty PM Campbell BI Wilson J Galvan E Berardi J Kleiner SM Kreider RB Stout JR Ziegenfuss T Spano M Smith A Antonio J International Society of Sports Nutrition position stand: meal frequency J Int Soc Sports Nutr 2011 8 4 10.1186/1550-2783-8-4 21410984 PMC3070624

[B109] Varady KA Intermittent versus daily calorie restriction: which diet regimen is more effective for weight loss? Obes Rev 2011 12 e593 e601 10.1111/j.1467-789X.2011.00873.x 21410865

[B110] Bosy-Westphal A Later W Hitze B Sato T Kossel E Gluer CC Heller M Muller MJ Accuracy of bioelectrical impedance consumer devices for measurement of body composition in comparison to whole body magnetic resonance imaging and dual X-ray absorptiometry Obes Facts 2008 1 319 324 20054195 10.1159/000176061 PMC6452160

[B111] Pateyjohns IR Brinkworth GD Buckley JD Noakes M Clifton PM Comparison of three bioelectrical impedance methods with DXA in overweight and obese men Obesity (Silver Spring) 2006 14 2064 2070 10.1038/oby.2006.241 17135624

[B112] Neovius M Hemmingsson E Freyschuss B Udden J Bioelectrical impedance underestimates total and truncal fatness in abdominally obese women Obesity (Silver Spring) 2006 14 1731 1738 10.1038/oby.2006.199 17062802

[B113] Stote KS Baer DJ Spears K Paul DR Harris GK Rumpler WV Strycula P Najjar SS Ferrucci L Ingram DK Longo DL Mattson MP A controlled trial of reduced meal frequency without caloric restriction in healthy, normal-weight, middle-aged adults Am J Clin Nutr 2007 85 981 988 17413096 10.1093/ajcn/85.4.981 PMC2645638

[B114] Iwao S Mori K Sato Y Effects of meal frequency on body composition during weight control in boxers Scand J Med Sci Sports 1996 6 265 272 8960647 10.1111/j.1600-0838.1996.tb00469.x

[B115] Benardot D Martin DE Thompson WR Roman SB Between-meal energy intake effects on body composition, performance, and totol caloric consumption in athletes Med Sci Sports Exerc 2005 37 S339

[B116] Norton LE Wilson GJ Optimal protein intake to maximize muscle protein synthesis: examinations of optimal meal protein intake Agro Food Industry Hi-Tech 2009 20 54 57

[B117] Bohe J Low JF Wolfe RR Rennie MJ Latency and duration of stimulation of human muscle protein synthesis during continuous infusion of amino acids J Physiol 2001 532 575 579 10.1111/j.1469-7793.2001.0575f.x 11306673 PMC2278544

[B118] Atherton PJ Etheridge T Watt PW Wilkinson D Selby A Rankin D Smith K Rennie MJ Muscle full effect after oral protein: time-dependent concordance and discordance between human muscle protein synthesis and mTORC1 signaling Am J Clin Nutr 2010 92 1080 1088 10.3945/ajcn.2010.29819 20844073

[B119] Munsters MJ Saris WH Effects of meal frequency on metabolic profiles and substrate partitioning in lean healthy males PLoS One 2012 7 e38632 10.1371/journal.pone.0038632 22719910 PMC3374835

[B120] Holmstrup M Owens CM Fairchild TJ Kanaley JA Effect of meal freqnency on glucose and insulin excursions over the course of a day Eur e-J Clin Nutr Metab 2010 5 277 280 10.1016/j.eclnm.2010.10.001

[B121] Leidy HJ Armstrong CL Tang M Mattes RD Campbell WW The influence of higher protein intake and greater eating frequency on appetite control in overweight and obese men Obesity (Silver Spring) 2010 18 1725 1732 10.1038/oby.2010.45 20339363 PMC4034047

[B122] Arciero PJ Ormsbee MJ Gentile CL Nindl BC Brestoff JR Ruby M Increased protein intake and meal frequency reduces abdominal fat during energy balance and energy deficit Obesity (Silver Spring) 2013 21 1357 1366 10.1002/oby.20296 23703835

[B123] Adechian S Balage M Remond D Migne C Quignard-Boulange A Marset-Baglieri A Rousset S Boirie Y Gaudichon C Dardevet D Mosoni L Protein feeding pattern, casein feeding or milk soluble protein feeding did not change the evolution of body composition during a short-term weight loss program Am J Physiol Endocrinol Metab 2012 303 E973 E982 10.1152/ajpendo.00285.2012 22895782

[B124] Moore DR Areta J Coffey VG Stellingwerff T Phillips SM Burke LM Cleroux M Godin JP Hawley JA Daytime pattern of post-exercise protein intake affects whole-body protein turnover in resistance-trained males Nutr Metab (Lond) 2012 9 91 10.1186/1743-7075-9-91 23067428 PMC3514209

[B125] Areta JL Burke LM Ross ML Camera DM West DW Broad EM Jeacocke NA Moore DR Stellingwerff T Phillips SM Hawley JA Coffey VG Timing and distribution of protein ingestion during prolonged recovery from resistance exercise alters myofibrillar protein synthesis J Physiol 2013 591 2319 2331 23459753 10.1113/jphysiol.2012.244897 PMC3650697

[B126] OCB/NANBF/IFPA Drug Testing Guidelines [http://www.thenaturalmusclenetwork.com/OCB/forms/DrugTestingGuidelines.pdf]

[B127] Campbell B Kreider RB Ziegenfuss T La Bounty P Roberts M Burke D Landis J Lopez H Antonio J International Society of Sports Nutrition position stand: protein and exercise J Int Soc Sports Nutr 2007 4 8 10.1186/1550-2783-4-8 17908291 PMC2117006

[B128] Buford TW Kreider RB Stout JR Greenwood M Campbell B Spano M Ziegenfuss T Lopez H Landis J Antonio J International Society of Sports Nutrition position stand: creatine supplementation and exercise J Int Soc Sports Nutr 2007 4 6 10.1186/1550-2783-4-6 17908288 PMC2048496

[B129] Kim H Kim C Carpentier A Poortmans J Studies on the safety of creatine supplementation Amino Acids 2011 40 1409 1418 10.1007/s00726-011-0878-2 21399917

[B130] Becque MD Lochmann JD Melrose DR Effects of oral creatine supplementation on muscular strength and body composition Med Sci Sports Exerc 2000 32 654 658 10.1097/00005768-200003000-00016 10731009

[B131] Volek JS Duncan ND Mazzetti SA Staron RS Putukian M Gomez AL Pearson DR Fink WJ Kraemer WJ Performance and muscle fiber adaptations to creatine supplementation and heavy resistance training Med Sci Sports Exerc 1999 31 1147 1156 10.1097/00005768-199908000-00011 10449017

[B132] Willoughby DS Rosene J Effects of oral creatine and resistance training on myosin heavy chain expression Med Sci Sports Exerc 2001 33 1674 1681 10.1097/00005768-200110000-00010 11581551

[B133] Vandenberghe K Goris M Van Hecke P Van Leemputte M Vangerven L Hespel P Long-term creatine intake is beneficial to muscle performance during resistance training J Appl Physiol 1997 83 2055 2063 9390981 10.1152/jappl.1997.83.6.2055

[B134] Stone MH Sanborn K Smith LL O'Bryant HS Hoke T Utter AC Johnson RL Boros R Hruby J Pierce KC Stone ME Garner B Effects of in-season (5 weeks) creatine and pyruvate supplementation on anaerobic performance and body composition in American football players Int J Sport Nutr 1999 9 146 165 10362452 10.1123/ijsn.9.2.146

[B135] Persky AM Brazeau GA Clinical pharmacology of the dietary supplement creatine monohydrate Pharmacol Rev 2001 53 161 176 11356982

[B136] Hultman E Soderlund K Timmons JA Cederblad G Greenhaff PL Muscle creatine loading in men J Appl Physiol 1996 81 232 237 8828669 10.1152/jappl.1996.81.1.232

[B137] Tallon MJ Child R Kre-alkalyn suppplementation has no beneficial effect on creatine-to-creatinine conversion rates Book Kre-alkalyn suppplementation has no beneficial effect on creatine-to-creatinine conversion rates 2007 City

[B138] Child RT MJ Creatine ethyl ester rapidly degrades to creatinine in stomach acid Book Creatine ethyl ester rapidly degrades to creatinine in stomach acid 2007

[B139] Spillane M Schoch R Cooke M Harvey T Greenwood M Kreider R Willoughby DS The effects of creatine ethyl ester supplementation combined with heavy resistance training on body composition, muscle performance, and serum and muscle creatine levels J Int Soc Sports Nutr 2009 6 6 10.1186/1550-2783-6-6 19228401 PMC2649889

[B140] Jagim AR Oliver JM Sanchez A Galvan E Fluckey J Riechman S Greenwood M Kelly K Meininger C Rasmussen C Kreider RB A buffered form of creatine does not promote greater changes in muscle creatine content, body composition, or training adaptations than creatine monohydrate J Int Soc Sports Nutr 2012 9 43 10.1186/1550-2783-9-43 22971354 PMC3479057

[B141] Artioli GG Gualano B Smith A Stout J Lancha AH Jr Role of beta-alanine supplementation on muscle carnosine and exercise performance Med Sci Sports Exerc 2010 42 1162 1173 20479615 10.1249/MSS.0b013e3181c74e38

[B142] Harris RC Tallon MJ Dunnett M Boobis L Coakley J Kim HJ Fallowfield JL Hill CA Sale C Wise JA The absorption of orally supplied beta-alanine and its effect on muscle carnosine synthesis in human vastus lateralis Amino Acids 2006 30 279 289 10.1007/s00726-006-0299-9 16554972

[B143] Derave W Ozdemir MS Harris RC Pottier A Reyngoudt H Koppo K Wise JA Achten E beta-Alanine supplementation augments muscle carnosine content and attenuates fatigue during repeated isokinetic contraction bouts in trained sprinters J Appl Physiol 2007 103 1736 1743 10.1152/japplphysiol.00397.2007 17690198

[B144] Hill CA Harris RC Kim HJ Harris BD Sale C Boobis LH Kim CK Wise JA Influence of beta-alanine supplementation on skeletal muscle carnosine concentrations and high intensity cycling capacity Amino Acids 2007 32 225 233 10.1007/s00726-006-0364-4 16868650

[B145] Van Thienen R Van Proeyen K Vanden Eynde P Puype J Lefere T Hespel P Beta-alanine improves sprint performance in endurance cycling Med Sci Sports Exerc 2009 41 898 903 10.1249/MSS.0b013e31818db708 19276843

[B146] Sale C Saunders B Hudson S Wise JA Harris RC Sunderland CD Effect of beta-alanine plus sodium bicarbonate on high-intensity cycling capacity Med Sci Sports Exerc 2011 43 1972 1978 21407127 10.1249/MSS.0b013e3182188501

[B147] Smith AE Walter AA Graef JL Kendall KL Moon JR Lockwood CM Fukuda DH Beck TW Cramer JT Stout JR Effects of beta-alanine supplementation and high-intensity interval training on endurance performance and body composition in men; a double-blind trial J Int Soc Sports Nutr 2009 6 5 10.1186/1550-2783-6-5 19210788 PMC2649036

[B148] Stout JR Cramer JT Zoeller RF Torok D Costa P Hoffman JR Harris RC O'Kroy J Effects of beta-alanine supplementation on the onset of neuromuscular fatigue and ventilatory threshold in women Amino Acids 2007 32 381 386 10.1007/s00726-006-0474-z 17136505

[B149] Hoffman J Ratamess NA Ross R Kang J Magrelli J Neese K Faigenbaum AD Wise JA Beta-alanine and the hormonal response to exercise Int J Sports Med 2008 29 952 958 10.1055/s-2008-1038678 18548362

[B150] Hoffman JR Ratamess NA Faigenbaum AD Ross R Kang J Stout JR Wise JA Short-duration beta-alanine supplementation increases training volume and reduces subjective feelings of fatigue in college football players Nutr Res 2008 28 31 35 10.1016/j.nutres.2007.11.004 19083385

[B151] Zoeller RF Stout JR O'Kroy JA Torok DJ Mielke M Effects of 28 days of beta-alanine and creatine monohydrate supplementation on aerobic power, ventilatory and lactate thresholds, and time to exhaustion Amino Acids 2007 33 505 510 10.1007/s00726-006-0399-6 16953366

[B152] Hoffman J Ratamess N Kang J Mangine G Faigenbaum A Stout J Effect of creatine and beta-alanine supplementation on performance and endocrine responses in strength/power athletes Int J Sport Nutr Exerc Metab 2006 16 430 446 17136944 10.1123/ijsnem.16.4.430

[B153] Kendrick IP Harris RC Kim HJ Kim CK Dang VH Lam TQ Bui TT Smith M Wise JA The effects of 10 weeks of resistance training combined with beta-alanine supplementation on whole body strength, force production, muscular endurance and body composition Amino Acids 2008 34 547 554 10.1007/s00726-007-0008-3 18175046

[B154] Sweeney KM Wright GA Glenn Brice A Doberstein ST The effect of beta-alanine supplementation on power performance during repeated sprint activity J Strength Cond Res 2010 24 79 87 10.1519/JSC.0b013e3181c63bd5 19935102

[B155] Hobson RM Saunders B Ball G Harris RC Sale C Effects of beta-alanine supplementation on exercise performance: a meta-analysis. Amino Acids 2012 43 25 37 10.1007/s00726-011-1200-z 22270875 PMC3374095

[B156] Lu P Xu W Sturman JA Dietary beta-alanine results in taurine depletion and cerebellar damage in adult cats J Neurosci Res 1996 43 112 119 10.1002/jnr.490430115 8838582

[B157] Smith HJ Mukerji P Tisdale MJ Attenuation of proteasome-induced proteolysis in skeletal muscle by {beta}-hydroxy-{beta}-methylbutyrate in cancer-induced muscle loss Cancer Res 2005 65 277 283 15665304

[B158] Eley HL Russell ST Baxter JH Mukerji P Tisdale MJ Signaling pathways initiated by beta-hydroxy-beta-methylbutyrate to attenuate the depression of protein synthesis in skeletal muscle in response to cachectic stimuli Am J Physiol Endocrinol Metab 2007 293 E923 E931 10.1152/ajpendo.00314.2007 17609254

[B159] Rathmacher JA Nissen S Panton L Clark RH Eubanks May P Barber AE D'Olimpio J Abumrad NN Supplementation with a combination of beta-hydroxy-beta-methylbutyrate (HMB), arginine, and glutamine is safe and could improve hematological parameters JPEN J Parenter Enteral Nutr 2004 28 65 75 10.1177/014860710402800265 15080599

[B160] Nissen S Sharp RL Panton L Vukovich M Trappe S Fuller JC Jr beta-hydroxy-beta-methylbutyrate (HMB) supplementation in humans is safe and may decrease cardiovascular risk factors J Nutr 2000 130 1937 1945 10917905 10.1093/jn/130.8.1937

[B161] Gallagher PM Carrithers JA Godard MP Schulze KE Trappe SW Beta-hydroxy-beta-methylbutyrate ingestion, part II: effects on hematology, hepatic and renal function Med Sci Sports Exerc 2000 32 2116 2119 10.1097/00005768-200012000-00023 11128860

[B162] Fitschen PJ Wilson GJ Wilson JM Wilund KR Efficacy of beta-hydroxy-beta-methylbutyrate supplementation in elderly and clinical populations Nutrition 2013 29 29 36 10.1016/j.nut.2012.05.005 23085015

[B163] Wilson GJ Wilson JM Manninen AH Effects of beta-hydroxy-beta-methylbutyrate (HMB) on exercise performance and body composition across varying levels of age, sex, and training experience: a review Nutr Metab (Lond) 2008 5 1 10.1186/1743-7075-5-1 18173841 PMC2245953

[B164] Wilson J Fitschen P Campbell B Wilson G Zanchi N Taylor L Wilborn C Kalman D Stout J Hoffman J Ziegenfuss T Lopez H Kreider R Smith-Ryan A Antonio J International Society of Sports Nutrition Position Stand: beta-hydroxy-beta-methylbutyrate (HMB) J Int Soc Sports Nutr 2013 10 6 10.1186/1550-2783-10-6 23374455 PMC3568064

[B165] Shimomura Y Yamamoto Y Bajotto G Sato J Murakami T Shimomura N Kobayashi H Mawatari K Nutraceutical effects of branched-chain amino acids on skeletal muscle J Nutr 2006 136 529S 532S 16424141 10.1093/jn/136.2.529S

[B166] Garlick PJ Grant I Amino acid infusion increases the sensitivity of muscle protein synthesis in vivo to insulin. Effect of branched-chain amino acids Biochem J 1988 254 579 584 3052439 10.1042/bj2540579 PMC1135117

[B167] Balage M Dardevet D Long-term effects of leucine supplementation on body composition Curr Opin Clin Nutr Metab Care 2010 13 265 270 10.1097/MCO.0b013e328336f6b8 20110810

[B168] Pencharz PB Elango R Ball RO Determination of the tolerable upper intake level of leucine in adult men J Nutr 2012 142 2220S 2224S 10.3945/jn.112.160259 23077191

[B169] Biolo G Tipton KD Klein S Wolfe RR An abundant supply of amino acids enhances the metabolic effect of exercise on muscle protein Am J Physiol 1997 273 E122 E129 9252488 10.1152/ajpendo.1997.273.1.E122

[B170] Tipton KD Ferrando AA Phillips SM Doyle D Jr Wolfe RR Postexercise net protein synthesis in human muscle from orally administered amino acids Am J Physiol 1999 276 E628 E634 10198297 10.1152/ajpendo.1999.276.4.E628

[B171] Louard RJ Barrett EJ Gelfand RA Effect of infused branched-chain amino acids on muscle and whole-body amino acid metabolism in man Clin Sci 1990 79 457 466 2174312 10.1042/cs0790457

[B172] Borsheim E Tipton KD Wolf SE Wolfe RR Essential amino acids and muscle protein recovery from resistance exercise Am J Physiol Endocrinol Metab 2002 283 E648 E657 12217881 10.1152/ajpendo.00466.2001

[B173] Stoppani J Scheett T Pena J Rudolph C Charlebois D Consuming a supplement containing branched-chain amino acids during a resistance-traning program increases lean mass, muscle strength, and fat loss J Int Soc Sports Nutr 2009 6 P1 10.1186/1550-2783-6-S1-P1 19660093 PMC3313152

[B174] Wilson GJ Layman DK Moulton CJ Norton LE Anthony TG Proud CG Rupassara SI Garlick PJ Leucine or carbohydrate supplementation reduces AMPK and eEF2 phosphorylation and extends postprandial muscle protein synthesis in rats Am J Physiol Endocrinol Metab 2011 301 E1236 E1242 10.1152/ajpendo.00242.2011 21917636 PMC4395871

[B175] Suryawan A Hawes JW Harris RA Shimomura Y Jenkins AE Hutson SM A molecular model of human branched-chain amino acid metabolism Am J Clin Nutr 1998 68 72 81 9665099 10.1093/ajcn/68.1.72

[B176] Fahs CA Heffernan KS Fernhall B Hemodynamic and vascular response to resistance exercise with L-arginine Med Sci Sports Exerc 2009 41 773 779 10.1249/MSS.0b013e3181909d9d 19276857

[B177] Tang JE Lysecki PJ Manolakos JJ MacDonald MJ Tarnopolsky MA Phillips SM Bolus arginine supplementation affects neither muscle blood flow nor muscle protein synthesis in young men at rest or after resistance exercise J Nutr 2011 141 195 200 10.3945/jn.110.130138 21191143

[B178] Volpi E Kobayashi H Sheffield-Moore M Mittendorfer B Wolfe RR Essential amino acids are primarily responsible for the amino acid stimulation of muscle protein anabolism in healthy elderly adults Am J Clin Nutr 2003 78 250 258 12885705 10.1093/ajcn/78.2.250 PMC3192452

[B179] Alvares TS Meirelles CM Bhambhani YN Paschoalin VM Gomes PS L-Arginine as a potential ergogenic aid in healthy subjects Sports Med 2011 41 233 248 10.2165/11538590-000000000-00000 21395365

[B180] Greer BK Jones BT Acute arginine supplementation fails to improve muscle endurance or affect blood pressure responses to resistance training J Strength Cond Res 2011 25 1789 1794 10.1519/JSC.0b013e3181e07569 21399536

[B181] McConell GK Effects of L-arginine supplementation on exercise metabolism Curr Opin Clin Nutr Metab Care 2007 10 46 51 10.1097/MCO.0b013e32801162fa 17143054

[B182] Shao A Hathcock JN Risk assessment for the amino acids taurine, L-glutamine and L-arginine Regul Toxicol Pharmacol 2008 50 376 399 10.1016/j.yrtph.2008.01.004 18325648

[B183] Perez-Guisado J Jakeman PM Citrulline malate enhances athletic anaerobic performance and relieves muscle soreness J Strength Cond Res 2010 24 1215 1222 10.1519/JSC.0b013e3181cb28e0 20386132

[B184] Bendahan D Mattei JP Ghattas B Confort-Gouny S Le Guern ME Cozzone PJ Citrulline/malate promotes aerobic energy production in human exercising muscle Br J Sports Med 2002 36 282 289 10.1136/bjsm.36.4.282 12145119 PMC1724533

[B185] Sureda A Cordova A Ferrer MD Perez G Tur JA Pons A L-citrulline-malate influence over branched chain amino acid utilization during exercise Eur J Appl Physiol 2010 110 341 351 10.1007/s00421-010-1509-4 20499249

[B186] Hickner RC Tanner CJ Evans CA Clark PD Haddock A Fortune C Geddis H Waugh W McCammon M L-citrulline reduces time to exhaustion and insulin response to a graded exercise test Med Sci Sports Exerc 2006 38 660 666 10.1249/01.mss.0000210197.02576.da 16679980

[B187] Gleeson M Dosing and efficacy of glutamine supplementation in human exercise and sport training J Nutr 2008 138 2045S 2049S 18806122 10.1093/jn/138.10.2045S

[B188] Antonio J Sanders MS Kalman D Woodgate D Street C The effects of high-dose glutamine ingestion on weightlifting performance J Strength Cond Res 2002 16 157 160 11834123

[B189] Haub MD Potteiger JA Nau KL Webster MJ Zebas CJ Acute L-glutamine ingestion does not improve maximal effort exercise J Sports Med Phys Fitness 1998 38 240 244 9830832

[B190] Colker CM Swain MA Fabrucini B Shi Q Kalman DS Effects of supplemental protein on body composition and muscular strength in healthy athletic male adults Curr Ther Res 2000 61 19 28 10.1016/S0011-393X(00)88492-1

[B191] Candow DG Chilibeck PD Burke DG Davison KS Smith-Palmer T Effect of glutamine supplementation combined with resistance training in young adults Eur J Appl Physiol 2001 86 142 149 10.1007/s00421-001-0523-y 11822473

[B192] Camilleri M Madsen K Spiller R Van Meerveld BG Verne GN Intestinal barrier function in health and gastrointestinal disease Neurogastroenterol Motil 2012 24 503 512 10.1111/j.1365-2982.2012.01921.x 22583600 PMC5595063

[B193] Ivy JL Kammer L Ding Z Wang B Bernard JR Liao YH Hwang J Improved cycling time-trial performance after ingestion of a caffeine energy drink Int J Sport Nutr Exerc Metab 2009 19 61 78 19403954 10.1123/ijsnem.19.1.61

[B194] McNaughton LR Lovell RJ Siegler J Midgley AW Moore L Bentley DJ The effects of caffeine ingestion on time trial cycling performance Int J Sports Physiol Perform 2008 3 157 163 19208924 10.1123/ijspp.3.2.157

[B195] Carr A Dawson B Schneiker K Goodman C Lay B Effect of caffeine supplementation on repeated sprint running performance J Sports Med Phys Fitness 2008 48 472 478 18997650

[B196] Glaister M Howatson G Abraham CS Lockey RA Goodwin JE Foley P McInnes G Caffeine supplementation and multiple sprint running performance Med Sci Sports Exerc 2008 40 1835 1840 10.1249/MSS.0b013e31817a8ad2 18799995

[B197] Green JM Wickwire PJ McLester JR Gendle S Hudson G Pritchett RC Laurent CM Effects of caffeine on repetitions to failure and ratings of perceived exertion during resistance training Int J Sports Physiol Perform 2007 2 250 259 19168925 10.1123/ijspp.2.3.250

[B198] Woolf K Bidwell WK Carlson AG The effect of caffeine as an ergogenic aid in anaerobic exercise Int J Sport Nutr Exerc Metab 2008 18 412 429 18708685 10.1123/ijsnem.18.4.412

[B199] Duncan MJ Oxford SW The effect of caffeine ingestion on mood state and bench press performance to failure J Strength Cond Res 2011 25 178 185 10.1519/JSC.0b013e318201bddb 21157384

[B200] Williams AD Cribb PJ Cooke MB Hayes A The effect of ephedra and caffeine on maximal strength and power in resistance-trained athletes J Strength Cond Res 2008 22 464 470 10.1519/JSC.0b013e3181660320 18550961

[B201] Hendrix CR Housh TJ Mielke M Zuniga JM Camic CL Johnson GO Schmidt RJ Housh DJ Acute effects of a caffeine-containing supplement on bench press and leg extension strength and time to exhaustion during cycle ergometry J Strength Cond Res 2010 24 859 865 10.1519/JSC.0b013e3181ae7976 19834348

[B202] Nawrot P Jordan S Eastwood J Rotstein J Hugenholtz A Feeley M Effects of caffeine on human health Food Addit Contam 2003 20 1 30 12519715 10.1080/0265203021000007840

[B203] Tarnopolsky MA Atkinson SA MacDougall JD Sale DG Sutton JR Physiological responses to caffeine during endurance running in habitual caffeine users Med Sci Sports Exerc 1989 21 418 424 2674593

[B204] Bazzarre TL Kleiner SM Litchford MD Nutrient intake, body fat, and lipid profiles of competitive male and female bodybuilders J Am Coll Nutr 1990 9 136 142 10.1080/07315724.1990.10720362 2338462

[B205] Kleiner SM Bazzarre TL Ainsworth BE Nutritional status of nationally ranked elite bodybuilders Int J Sport Nutr 1994 4 54 69 8167655 10.1123/ijsn.4.1.54

[B206] Hickson JF Jr Johnson TE Lee W Sidor RJ Nutrition and the precontest preparations of a male bodybuilder J Am Diet Assoc 1990 90 264 267 2303663

[B207] Andersen RE Barlett SJ Morgan GD Brownell KD Weight loss, psychological, and nutritional patterns in competitive male body builders Int J Eat Disord 1995 18 49 57 10.1002/1098-108X(199507)18:1<49::AID-EAT2260180106>3.0.CO;2-C 7670443

[B208] Balon TW Horowitz JF Fitzsimmons KM Effects of carbohydrate loading and weight-lifting on muscle girth Int J Sport Nutr 1992 2 328 334 1299502 10.1123/ijsn.2.4.328

[B209] Costill DL Cote R Fink W Muscle water and electrolytes following varied levels of dehydration in man J Appl Physiol 1976 40 6 11 1248983 10.1152/jappl.1976.40.1.6

[B210] Goldfield GS Blouin AG Woodside DB Body image, binge eating, and bulimia nervosa in male bodybuilders Can J Psychiatry 2006 51 160 168 16618007 10.1177/070674370605100306

[B211] Mangweth B Pope HG Jr Kemmler G Ebenbichler C Hausmann A De Col C Kreutner B Kinzl J Biebl W Body image and psychopathology in male bodybuilders Psychother Psychosom 2001 70 38 43 10.1159/000056223 11150937

[B212] Baghurst T Lirgg C Characteristics of muscle dysmorphia in male football, weight training, and competitive natural and non-natural bodybuilding samples Body Image 2009 6 221 227 10.1016/j.bodyim.2009.03.002 19410526

[B213] Pickett TC Lewis RJ Cash TF Men, muscles, and body image: comparisons of competitive bodybuilders, weight trainers, and athletically active controls Br J Sports Med 2005 39 217 222 discussion 217–222 10.1136/bjsm.2004.012013 15793091 PMC1725169

[B214] Jankauskiene R Kardelis K Pajaujiene S Muscle size satisfaction and predisposition for a health harmful practice in bodybuilders and recreational gymnasium users Medicina (Kaunas) 2007 43 338 346 17485962

[B215] Walberg JL Johnston CS Menstrual function and eating behavior in female recreational weight lifters and competitive body builders Med Sci Sports Exerc 1991 23 30 36 1997810

[B216] Sundgot-Borgen J Garthe I Elite athletes in aesthetic and Olympic weight-class sports and the challenge of body weight and body compositions J Sports Sci 2011 29 Suppl 1 S101 S114 21500080 10.1080/02640414.2011.565783

